# Nanoformulations in Pharmaceutical and Biomedical Applications: Green Perspectives

**DOI:** 10.3390/ijms25115842

**Published:** 2024-05-27

**Authors:** Sanja Petrovic, Bogdan Bita, Marcela-Elisabeta Barbinta-Patrascu

**Affiliations:** 1 Department of Chemical Technologies, Faculty of Technology, University of Nis, Bulevar Oslobodjenja 124, 16000 Leskovac, Serbia; milenkovic_sanja@yahoo.com; 2 Department of Electricity, Solid-State Physics and Biophysics, Faculty of Physics, University of Bucharest, 405 Atomistilor Street, P.O. Box MG-11, 077125 Magurele, Romania; bogdan.bita@fizica.unibuc.ro

**Keywords:** nano-therapy, nanopharmaceuticals carriers, biomaterials, photo-nanotheranostics, biomimetics, bioinspiration, cyborg cells, green design

## Abstract

This study provides a brief discussion of the major nanopharmaceuticals formulations as well as the impact of nanotechnology on the future of pharmaceuticals. Effective and eco-friendly strategies of biofabrication are also highlighted. Modern approaches to designing pharmaceutical nanoformulations (e.g., 3D printing, Phyto-Nanotechnology, Biomimetics/Bioinspiration, etc.) are outlined. This paper discusses the need to use natural resources for the “green” design of new nanoformulations with therapeutic efficiency. Nanopharmaceuticals research is still in its early stages, and the preparation of nanomaterials must be carefully considered. Therefore, safety and long-term effects of pharmaceutical nanoformulations must not be overlooked. The testing of nanopharmaceuticals represents an essential point in their further applications. Vegetal scaffolds obtained by decellularizing plant leaves represent a valuable, bioinspired model for nanopharmaceutical testing that avoids using animals. Nanoformulations are critical in various fields, especially in pharmacy, medicine, agriculture, and material science, due to their unique properties and advantages over conventional formulations that allows improved solubility, bioavailability, targeted drug delivery, controlled release, and reduced toxicity. Nanopharmaceuticals have transitioned from experimental stages to being a vital component of clinical practice, significantly improving outcomes in medical fields for cancer treatment, infectious diseases, neurological disorders, personalized medicine, and advanced diagnostics. Here are the key points highlighting their importance. The significant challenges, opportunities, and future directions are mentioned in the final section.

## 1. Introduction

At the end of the 20th century, nanotechnology began to find applications in pharmacy and medicine [1]. The use of nanomaterials in these areas has led to the emergence of a new generation of products [2]. Nanotechnology enabled the understanding and control of matter at dimensions between approximately 1 and 100 nm, where unique phenomena enable novel applications which are not feasible when working with bulk materials or even with single atoms or molecules [3].

Numerous studies have focused on discovering and enhancing new medicines capable of precisely targeting disease sites. Nanotechnology plays a crucial role in facilitating the delivery of therapeutic agents to these sites by overcoming inherent limitations. Many drugs face challenges related to solubility, permeability, and bioavailability, which result in suboptimal pharmacokinetics, particularly concerning administration routes [4]. The objective is to design a dosage form with an effective pharmacokinetic delivery system. Nanoparticle-based drugs can function either as therapeutic agents themselves or as carriers for transporting diverse therapeutic agents to specific areas of the body [5,6,7].

Nanopharmaceuticals, leveraging the principles of nanotechnology, have revolutionized drug delivery systems and therapeutic strategies in modern medicine. By utilizing nanoparticles (NPs) and nanocarriers (NCs), these advanced formulations offer superior targeting capabilities, enhanced bioavailability, and reduced toxicity, addressing critical limitations of conventional therapies. The clinical success of various nanopharmaceuticals underscores their transformative impact on healthcare. The use of nanoparticles (NPs) in drug manufacturing offers numerous advantages over conventional controlled drug delivery systems, including: (1) the precise delivery of therapeutic agents to targeted tissues, reducing total dosage and potential toxic effects; (2) enhancing the stability and bioavailability of active pharmaceutical ingredients post-administration; (3) exhibiting superior safety and efficacy profiles; (4) enabling the controlled release of drugs over desired timeframes; (5) facilitating passive targeting and drug accumulation in malignant tumors and other pathological sites via the enhanced permeability and retention (EPR) effect; and (6) potentially rendering nanopharmaceutical products more cost-effective compared to traditional counterparts [8,9].

The current study offers insights into current nanopharmaceutical forms, highlighting diverse carrier types and applications. It presents a comprehensive overview of nanomedicine applications in preventing, diagnosing, and treating various diseases, such as cancer, infections, blood disorders, cardiovascular diseases, immune-related conditions, and nervous system disorders. Additionally, this review discusses important considerations and perspectives in this field.

Nanomedicine and nano-delivery systems represent a realm of burgeoning sciences with vast potential. In this domain, nanoscale carriers are harnessed to deliver therapeutic drugs precisely to targeted sites in a controlled manner. This approach offers numerous advantages, including enhanced efficacy and minimized adverse drug reactions. Recent investigations have extensively explored nanocarriers such as dendrimers, liposomes, nanotubes, and nanoparticles. Researchers have focused on their structural characteristics, size manipulation, and selective diagnosis using disease imaging molecules, ushering in a paradigm shift in drug delivery [10].

Nanopharmaceutical systems generally include products that may in their own right or in combination with another moiety bring some therapeutic benefit. They may also include engineered nanostructured systems that act as a drug carrier. Also, those can include a delivery vehicle or a delivery system for drugs and therapeutic agents, given the numerous nanosystems which have found application in diagnostics. Rivera and collaborators [11] have defined nanopharmaceuticals as: *pharmaceuticals engineered on the nanoscale, i.e., pharmaceuticals where the nanomaterial plays the pivotal therapeutic role or adds additional functionality to the previous compound*.

The three main uses of nanomaterials in the fields of medicine and pharmacy are in the areas of diagnostics, regenerative medicine, and therapy. The diagnostics include the possibility of applying nanomaterials as quantum dots for high-quality tissue screening, all the way to the production of multi-modal cameras and nanorobots that will capture tissue. In the field of regenerative medicine, special attention will be paid to nanotechnologies for skin and motor neurons, and in the field of therapy, the production of magnetic and paramagnetic nanoparticles as well as nanocarriers with active principles. An extensive examination of the scientific literature indicates that drug delivery using nanotechnology has yielded favorable outcomes in pain management. This approach not only restricts the side effects, but also enhances the efficacy of analgesic drugs. Beyond its drug delivery capabilities, nanotechnology has facilitated the design of advanced nanosystems that contribute to improved imaging and diagnostics. This advancement enables swift disease diagnosis, significantly influencing pain control. Additionally, the evolving tools in nanotechnology allow the precise management of pain, providing a means to assess the effectiveness of different interventions [12].

For instance, Doxil, a liposomal formulation of doxorubicin, has set a precedent in cancer treatment by significantly reducing cardiotoxicity and enhancing drug accumulation in tumors compared to free doxorubicin. Similarly, Abraxane, an albumin-bound paclitaxel, has demonstrated improved solubility and tumor targeting, leading to its approval for breast cancer, non-small cell lung cancer, and pancreatic cancer. The use of polymeric nanoparticles, as seen with Genexol-PM, a paclitaxel-loaded polymeric micelle, has enhanced the solubility and bioavailability of paclitaxel, leading to better therapeutic outcomes for metastatic breast cancer patients. In addition, Feraheme (ferumoxytol), an iron oxide nanoparticle, serves a dual purpose by acting as an iron replacement therapy and an MRI contrast agent, demonstrating the versatility of nanopharmaceuticals [13].

Nanosystems used in biomedical applications can be classified in different ways according to their elemental composition, size, structure, and function, or perhaps a structure–function relationship [14]. According to their composition, they can be organic or inorganic in nature, or hybrids. They can also be divided into liquids and solids according to their physical state. The division according to their size is proposed by Pokropivny and Skorokhod [15]: zero-dimensional, where all dimensions are less than 100 nm (0D—fullerenes, for example); one-dimensional (1D—nanofibers, nanotubes, nanowires, for example); two-dimensional (2D—nanoplates, for example); and three-dimensional (3D-dendrimers, for example). Nanomaterials can be formed directly from a basic material using some physical methods, such as photolithography, laser processing, and mechanical techniques, or they can be formed from molecular structures as a starting material, which, as a result, can produce new nanomaterials through various chemical reactions or physical treatments. In general, the most important difference between basic materials and nanomaterials is that nanomaterials have a small surface and a large number of atoms on the surface, which provide them with a pronounced surface energy and a large specific surface area per unit of mass [16]. That means nanomaterials have high reactivity, which means that they reduce mass, increase stability, and improve functionality. In the continuation of this current study, the most applied nanomaterials in the bio-pharmaceutical field will be presented.

Generally in pharmacies, the most convenient are nanostructured materials that represent processed forms of raw nanomaterials with special shapes or functionalities of a polymeric (fullerenes, fullerenols, and carbon nanotubes) or non-polymeric type (lipid-based nanoparticles, quantum dots, and metal nanoparticles).

**Figure 1:**
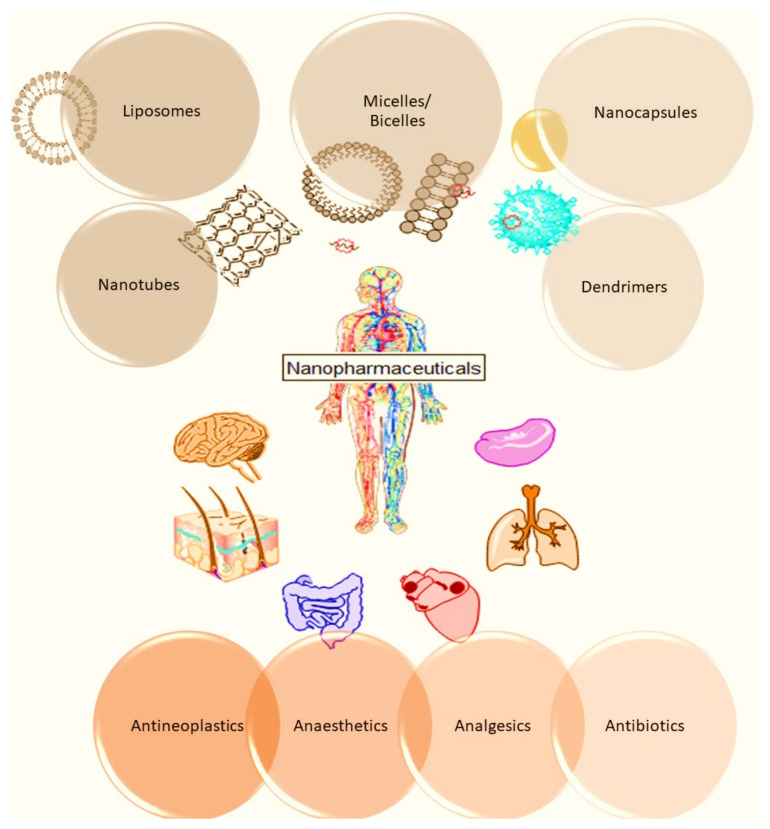
Illustration of wide use of nanopharmaceuticals. Figure was created with ChemOfficeUltra 2007 and with PowerPoint Version 2307.

Many nano-drugs such as antineoplastics, anaesthetics, analgesics, and antibiotics are the most common in the following pharmaceutical forms of powder infusions, solutions, suspensions for injections as well as emulsions, and they all can be classified as nanopharmaceuticals (Figure 1). Nanomaterials are particularly useful in delivering drugs to the tumor tissues. Due to their characteristics, nanopharmaceuticals have many advantages over conventional chemotherapy preparations. The biggest advantage is the targeted delivery of the drug to the tumor tissue, which results in reduced systemic toxicity, the most prominent disadvantage of conventional chemotherapy. Thanks to the clinically achieved success, in recent years, among all the nanocarriers, liposomes and micelles have achieved the most attention and the greatest application. Based on the administration routes by which nanopharmaceuticals can be applied, we can distinguish intravenous, intranasal, intracerebral, intrathecal, intraventricular, intraperitoneal, transcranial, oral, and ocular applications of nanomaterial forms.

Nanomaterials or nanoparticles are not always safe for use as their application could increase the health risk due to the side effects caused by classic drugs. However, if nanomaterials and nanoparticles meet the stated compatibility, then their application in biomedicine can be many times more beneficial than conventional drugs. Potential hazards of nanomaterials in the field of pharmacy will also be discussed in this study.

For the clinical success of the nanoformulations, crucial parameters such as fabrication strategies, physical properties, drug-loading efficiencies, drug release potential, and minimal carrier toxicity must be considered. Among these, lipid-based nanoparticles have the advantage of being the least toxic for *in vivo* applications, particularly in the realms of DNA/RNA and drug delivery [17].

Since the first globally marketed nanomedicines approved by the United States Food and Drug Administration (FDA) and the European Medicines Agency (EMA), the number of marketed nanopharmaceuticals showed an increasing trend [18].

These examples highlight the importance and efficacy of nanopharmaceuticals in clinical applications, driving the need for continued research and development in this field. The following study delves into the latest advancements and future directions of nanopharmaceuticals, aiming to further enhance their clinical utility and broaden their therapeutic scope. Specific examples of NPs and NCs are used to emphasize the clinical relevance and importance of nanopharmaceuticals. This sets the stage for discussing their advancements and potential in subsequent sections.

In the following sections, we will present the most used nanopharmaceuticals.

## 2. The Main Categories of Commonly Used Nanopharmaceuticals

### 2.1. Nanomicelles

Nanomicelles are structures around 5–100 nm that are able to take both hydrophilic and hydrophobic agents. They are formed when amphiphilic molecules assemble themselves under a critical micellar concentration—CMC. The particles may be formed in aqueous or non-aqueous solutions where the nonpolar region forms the interior and the polar region forms the exterior phase of a micelle. Depending on the ionic strength, surfactant concentration, and pH of the solutions, micelles usually have a spherical form. Still, sometimes, they can have a cylinder and ellipsoid shape too [19]. A number of surfactants in a solution and the chain length of surfactant molecules influence the micelle formation. If a molecule’s chain length is longer, then micelles will form at lower concentrations. Also, if dissolved salts are present in the solution, a lower critical micelle concentration is needed. Alcohols influence the CMC, with the CMC value increasing from methanol to butanol. Temperature increases also increase the CMC [20]. Micelles are usually made through surfactant molecules that may be nonionic, ionic, and cationic detergents. Some nanomicelles may contain a mixture of lipids and detergents. The CMC and the typical number of detergent molecules depend on the micelles’ lipids concentrations. Polymeric micelles formed by the self-assembling of amphiphilic block copolymers in an aqueous environment function as a solubilizing agent for hydrophobic drugs. It is the best alternative to enhance the solubility and bioavailability of the drugs loaded [21].

The micelle structures have disadvantages because of their inefficient drug-loading capabilities, poor physical *in vivo* stability, and insufficient cellular interactions with neutral micelles [22].

Various applications of nanomicelles for supporting human health are displayed in Table 1.

### 2.2. Lipid-Based Nanoparticles

The development of advanced lipid-based nanoparticles (LNPs) plays a crucial role in addressing complex biomedical questions and overcoming physiological challenges in the field of cancer nanomedicines. Since the FDA approved the first cancer nanomedicine in 1995, significant progress has been made in the design of smart nanomedicines. These advancements focus on functionalizing both the surfaces and interiors of LNPs to enhance therapeutic effects, achieve effective intratumoral distribution, and avoid rapid clearance and degradation *in vivo* [49].

Innovations in engineering lipid-based and hybrid lipid NPs (combining lipidic and polymeric components) have paved the way for co-delivery, tumor targeting, combination therapy, and cancer theranostics. The development of multifunctional nanoplatforms is a key strategy to address drug-resistant cancer cells and overcome barriers in delivering anticancer molecules [50].

Recent research has focused intensively on molecular and diagnostic imaging as a crucial aspect of treating various diseases. In medical imaging, several modalities, each with unique strengths, are employed, including Magnetic resonance imaging (MRI), ultrasound imaging, computed tomography (CT), positron emission tomography (PET), and single-photon emission computed tomography (SPECT). Specific contrast agents are essential for each of these systems to achieve optimal imaging quality. A review of Mirahadi et al. [51] explores the role of LNPs in medical diagnosis and imaging. Nanoparticles, particularly lipid-based ones like solid lipid nanoparticles (SLNs), nanostructured lipid carriers (NLCs), and liposomes, are innovative tools for researching and diagnosing various diseases, especially cancers. These lipid-based nanoparticles are preferred in imaging due to their advantageous properties.

Qizilbash et al. highlighted the role of lipid-based nanocarriers in the treatment of breast cancer [52]. Standard breast cancer treatments encompass surgery, chemotherapy, and radiotherapy. However, no singular treatment method has proven universally effective due to challenges like cancer stem cell metastasis and chemo-resistance. Consequently, using nanocarrier systems becomes crucial, particularly for targeting breast cancer stem cells. The paper of Qizilbash et al. explores breast cancer treatment options, encompassing modern procedures like chemotherapy, and introduces innovative therapeutic approaches that emphasize the role of lipidic nanocarriers loaded with chemotherapeutic drugs. Nanoemulsions, SLNs, NLCs, and liposomes are investigated, demonstrating their potential in limiting cancer cell growth, reducing recurrence risk, and minimizing post-chemotherapy metastasis.

Despite significant advancements in understanding breast cancer pathogenesis, prognosis, diagnosis, and treatment, it continues to be a leading cause of female mortality globally. Chemotherapies, while effective, face critical limitations, notably their lack of specificity leading to systemic toxicity and the development of multi-drug resistance in cancer cells over time. Liposomes have emerged as a valuable drug delivery system. Still, only a few of the numerous liposomal systems developed each year have received clinical approval. None of them incorporate active targeting [53].

Some aspects related to liposomal nanoformulations are further presented.

### 2.3. Liposomes

One of the best choices when it comes to nanocarriers of pharmaceutical forms are certainly liposomes. Liposomes, minute vesicles composed of one or more lipid bilayers, serve as effective carriers for encapsulating hydrophilic, lipophilic, and amphiphilic biological active agents. These lipid vesicles are versatile and promising tools in the field of medicine, and they are used in treating various diseases [54], being used as drug carriers in the form of vesicles composed of lipids that are organized in one or more layers, forming the hydrophobic part of the liposome, while the inner aqueous media represent the hydrophilic parts which can include hydrophilic pharmaceutical forms. The frequent use of liposomes began in 1980, and even today they are one of the most common choices when it comes to carriers. There are several reasons for that, they are carriers of all types of compounds, hydrophilic, hydrophobic, and amphiphilic, and they are also safe to use. Unlike other carriers such as dendrimers, CNTs, and others, liposomes are organic carriers obtained from natural raw materials.

Liposomes can be made of saturated or unsaturated lipids of different chain lengths [55]. The choice of lipids depends on the incorporated substance itself—on its nature. The liposome phospholipid bilayer can create different possibilities for the final pharmaceutical forms that are incorporated precisely on the basis of the starting material, i.e., the nature of the phospholipids.

**Figure 2:**
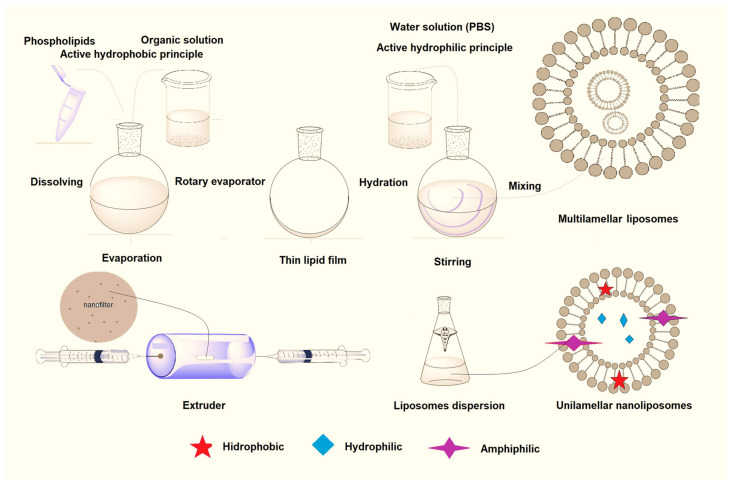
Schematic representation of liposome preparation by thin film hydration method. Figure was created with ChemOfficeUltra 2007 and with PowerPoint.

Since liposomes can be over 1000 nm in size, they cannot be considered nanomaterials, but liposomes with dimensions of 100 nm and less are so-called *nanoliposomes*. The methods of obtaining nanoliposomes can be different, and the choice of the manufacturing method will depend on the active component that the liposomes carry. Figure 2 illustrates the liposome preparation by the thin film hydration method, which involves two main stages: (1) the hydrophobic-lipid film preparation, and (2) the hydration of the obtained thin-lipid film. If the active principle to be loaded is lipid soluble, it will be included with the hydrophobic phase at the start of the preparation method. If not, it will be dissolved in the hydrophilic phase. The active principle must be kept at a temperature that is not degrading it, so the phase transition temperature of lipids until liposomal preparation must be consistent with the temperature at which the active component is stable. That is, the choice of lipid should be based on whether it corresponds to the active component, i.e., the temperature at which it is stable (for example, the DPPC lipid has a phase transition temperature of 42 °C and DMPC has 18 °C).

The preparation of liposomes requires the use of organic solvents. However, removing these solvents is time-consuming, and the residual solvent poses both hidden dangers and a threat to the stability of anionic liposomes. To address this issue, glycerol, a physiologically compatible substance that does not require removal, is employed to facilitate lipid dispersion and anionic liposome formation. Han et al. developed anionic liposomes without organic solvents for effective siRNA delivery [56]. This is a “green” approach that simplifies the preparation process and saves time.

The liposomes’ drawbacks are poor stability, poor series reproducibility, difficulties in sterilization, and a small drug-binding capacity. Various physical and chemical agents can damage the phospholipid bilayers of liposomes and also may alter the physicochemical properties of the liposomal drug contents. A (bio)polymeric coat can improve the liposomal stability, ensuring a sustained drugs release. Thus, chitosan (CS)-coated liposomes known as “chitosomes” are more stable nanocarriers than liposomes alone, and they are used especially in the transdermal delivery of bioactives [57]. Another research group [58] developed long-circulating liposomes decorated with DNA and then covered with an opsonin-deficient protein corona. This lipoplex called “proteoDNAsome” has a pronounced ability to evade capture by immune cells *in vivo*, being more bioperformant than PEGylated lipoplexes.

Today there is a remarkable molecular diversity of liposomes such as ufasomes, phytosomes, terpesomes, bilosomes, aspasomes, etc., [57] assured by their composition and by their coating. For example, if they are coated with poly(ethylene glycol) (PEG) (PEGylated liposomes) or with a combination of polyvinyl alcohol and PEG, the liposomes show a long-circulating function which is better in the latter case [58,59].

Moreover, liposomes have been used to prepare advanced cell-membrane-coated biomimetic nanocarriers to enhance the bioactivities of the therapeutical agents carried, as well as also offering long circulating times, simultaneous multidrug delivery, high biocompatibility, and targeting capabilities [60]. The cell-membrane-coated biomimetics system, which combines an isolated cell membrane with a nanocarrier, mimics the function of a cell, improves bio-interfacing skills, and protects the encapsulated drug by avoiding degradation [60]. Biomimetic nanoliposomes for cancer treatment were developed by coating liposomes with cell membranes derived from Red Blood Cells (RBCs), leukocytes, platelets, stem cells, bacteria, cancer cells, etc., [61]. By combining biomembranes from different cell types, novel Hybrid Cell-Membrane-Coated Liposomes with enhanced bioactivities were achieved [61]. For example, Xie et al. [61] have prepared a liposome-based delivery system by camouflaging liposomes with a hybrid cell membrane (RBC membrane and cancer cell membrane) and also the surface modification with RGD enabling the obtained nanoplatforms to be targeted at the tumor site. These hybrid membrane-coated liposomes presented good bioperformance (e.g., prolonged half-life, increased immune evasion, tumor targeting ability, and good antitumor activity without harming the healthy tissue). RGD is the tripeptide arginyl-glycyl-aspartic acid (Arg-Gly-Asp, R: arginine; G: glycine; D: aspartic acid) which was originally identified as the sequence within fibronectin that mediates cell attachment [62].

### 2.4. Dendrimers

Dendrimers are nano-sized polymeric macromolecules with a tree-like structure consisting of the core, branches, and terminal groups [63]. The three-dimensional geometric shape where the branches repeat around the central core enables the insertion of pharmaceutical and diagnostic agents. Dendrimers can be obtained by divergent or convergent synthesis, or through a series of controlled polymer reactions. Since dendrimers resemble spheres with countless cavities between branches, more than one active form can be inserted or placed. Dendrimers used in pharmacy for drug release and for diagnostic purposes usually have multiple functional groups on the surface and a diameter between 10 and 100 nm. The most important applications of dendrimers are gene therapy, immunosuppression, and contrast in MRI methods [64]. Due to the small dimensions of dendrimers, they are ideal carriers with a defined molecular weight and very low polydispersity index.

To address these challenges, nanomaterials, particularly dendrimers, are increasingly used in cancer therapies. Dendrimers, characterized by unique properties, can be precisely controlled during production to achieve the desired characteristics. These polymeric molecules play a crucial role in cancer diagnosis and treatment by facilitating the targeted distribution of pharmacological substances [65]. Dendrimers exhibit the capability to achieve various objectives in anticancer therapy simultaneously. This includes targeted delivery to tumor cells and spare healthy tissue, the controlled release of anticancer agents within the tumor microenvironment, and the integration of multiple anticancer strategies. These strategies encompass administering anticancer molecules to enhance their effectiveness through methods like photothermal therapy (PTT) or photodynamic therapy (PDT) [66].

### 2.5. Carbon Nanomaterials in Nanopharmaceutical Applications

Between carbon nanomaterials (CNMs), the most used in nanopharmaceutical applications are fullerenes and carbon nanotubes (CNTs) which are carbon allotropes that have a unique architecture with a hollow structure, allowing the therapeutic agents to be attached inside or on their surface.

#### 2.5.1. Fullerenes

Fullerenes are a special type of molecule built from pure carbon in the form of a closed sphere. Fullerene C60 contains 60 C-atoms arranged in a pattern of interconnected hexagons and pentagons, known as a truncated icosahedron [67]. Fullerene configurations of a less regular spherical shape are C20, C36, C70, and C78. Fullerenes represent relatively large systems with numerous positive properties, like antioxidant activity (elimination of free radicals—ROS), the suppression of metastasis, treatment of Alzheimer’s and Parkinson’s diseases, and treatment of hepatitis C and HIV infection [57,68,69]. Fullerenols as fullerene polyhydroxy derivatives are the most effective antioxidants thanks to the electrochemical properties that allow the reaction with ROS such as superoxide (O^.2−^) and hydroxyl radicals (•OH), where the center of the fullerene molecules acts as a sponge for free radicals. Chemical surface modifications of fullerenes by OH groups give different solubility properties and antioxidant activity in an aqueous medium. By changing the solvent, temperature, C60 concentration, and mixing process, the size, structure, and charge of C60 are affected, as well as the final properties of nanoparticles [70]. Fullerenes and their derivatives are used in the diagnosis and treatment of tumors [70,71]. The fullerenes undergo photoexcitation upon illumination; therefore, they are used in PDT for the destruction of various cancer cells.

#### 2.5.2. Carbon Nanotubes (CNTs)

The unique structural characteristics of carbon nanoparticles, such as their elongated shape and ability to be modified and used as carrier vectors, along with their potential to be compatible with living organisms, make them valuable for delivering pharmaceuticals at the nanoscale. CNTs have the additional benefit of serving as prospective nanofluidic devices for precise medication administration [72].

CNTs look like microsyringes due to their very high value of the L/d ratio; therefore, they can cross the biological barriers, allowing their drug delivery applications [73]. This particular geometry gives CNTs the possibility to enter living cells without causing cell death or damage [73,74].

Thus, once inside living cells, CNTs release the drugs. However, some limitations of using carbon-based nanomaterials in bio-pharmaceutical applications include their aggregation tendency and toxicity due to geometrical parameters, surface functionalization, and residuals arising from the fabrication process. To overcome these drawbacks, the surface of carbon nanomaterials must be functionalized with biomolecules or with biomimetic cell membranes. In addition, it must adopt “green” strategies for the synthesis of carbon nanomaterials. For example, natural precursors such as neem oil, eucalyptus oil, coconut oil, camphor, and plant extracts (e.g., rose, garden grass, walnut, and neem) are used for the “green” synthesis of carbon nanomaterials [74].

#### 2.5.3. Carbon Nanohorns

Carbon nanohorns or carbon nanocons (CNHs) are cylindrical structures analogous to CNTs except that they are closed at one end, forming a “horn” (a cone-shaped cap). CNHs tend to form spherical dahlia-flowerlike aggregates with a size of less than 100 nm [75,76]. The individual CNHs extend outward from the surface of the aggregate, resembling the petals of a dahlia. There are 13 individual CNHs, cone-shaped structures made of graphitic carbon. CNTs have precise diameters that can be controlled. In contrast, CNHs have larger diameters as their length increases, due to the expanding base of the nanocone [76]. CNHs have been used in the delivery of various drugs such as dexamethasone, prednisolone, doxorubicin, and cisplatin. Interestingly, pristine CNHs have been exploited as effective nanotherapeutics in cancer therapy, being often used alone without drugs due to their ability to promote photothermal cancer cell ablation [5].

### 2.6. Metal-Based Nanopharmaceuticals

Metal-based nanopharmaceuticals have been used in a wide range of applications including drug delivery and diagnosis [77]. A special attention has been given to metallic nanoparticles (MNPs), metal oxide nanoparticles (MONPs), quantum dots (QD)s, and metal–organic frameworks (MOFs).

#### 2.6.1. Metal and Metal Oxide Nanoparticles

The most commonly used nanomaterials are MNPs and MONPs, due to their small size and large surface area, properties that allow novel ways of diagnosing and treating diseases [78].

Magnetic nanoparticles like iron, nickel, and cobalt can be manipulated by using magnetic fields. Due to their good conductivity, high chemical stability, good catalytic activity, and efficient antibacterial activity, they are used in a wide range of applications such as catalysis, electronics, photonics, optoelectronics, information technology, sensing, and medicine [79,80]. Magnetic iron oxide nanoparticles have numerous applications in diagnosis, MRI, multimodal imaging, chemotherapy, hyperthermal therapy, photodynamic therapy, and gene delivery [81,82]. Metal-based nanocomposites are widely used in various biomedical applications due to their unique properties as well. Significant attention is given to the design of magnetoplasmonic nanohybrids, which exploit synergistic properties for biomedical applications. A straightforward method was used to prepare plasmonic magnetic Au-MnO heterostructured hybrid nanoparticles for the imaging-guided photothermal therapy of cancers *in vitro*. This approach aims to mitigate the serious drawbacks of chemotherapy and gadolinium-based contrast agents [83]. Designing magnetic hydroxyapatite (MHAp) nanoparticles with optimal dimensions, stability, and biocompatibility for specific biomedical applications remains an emerging challenge. A straightforward approach to prepare ROS-responsive chlorin e6 (Ce6) and silk fibroin-loaded ultrathin magnetic hydroxyapatite nanorods (MHNRs) for T1-magnetic resonance imaging and photodynamic therapy has been made. Specifically, Fe_3_O_4_-HAp nanorods were synthesized using the hydrothermal method. Pluronic^®^ F-127 was then utilized to enhance aqueous dispersion, biocompatibility, and loading capacity. Additionally, silk fibroin protein was encapsulated with a triblock copolymer, achieving high-loading efficiency. The theranostic nano-assembly, MHNRs-SF-Ce6, was completed by incorporating Ce6. The prepared MHNRs demonstrated excellent biocompatibility, cellular uptake, and ROS generation capability *in vitro* following 660 nm laser irradiation, which induced apoptosis in 4T1 mouse breast cancer cells [84].

Oral drug nanoformulations based on magnetic nanoparticles (usually made of ferrite, magnetic materials, or iron oxide) have been used as nanocarriers for drug localization, controlled release, and targeted delivery to the gastrointestinal (GI) tract. Their surface can be modified with ligands (peptides or small-molecule ligands) in such a way as to achieve targeting, thus reducing their impact on non-targeted tissues [82]. Magnetic nanoparticles were functionalized with the common anticancer drugs 5-Fluorouracil (5-FU), irinotecan, and oxaliplatin for the treatment of colon cancer.

The poly(acrylic acid) (PAA)-protected platinum nanoparticles (PAA-Pt) proved to be efficient scavengers of free radicals like peroxyl radicals, superoxide anions, and hydroxyl radicals [85]. An imbalanced oxidative status due to the production of ROS can be reduced by the presence of antioxidants such as PtNPs [86]. The research of Watanabe, et al. demonstrated that these nanoparticles can be used in the treatment of diseases related to oxidative stress and ageing because these platinum nanoparticles have activity like mitochondrial electron transfer complex I [85]. Moreover, these PtNPs act as a Superoxide dismutase (SOD)/catalase mimetic system—the so-called “nanozyme” which are artificial metalloenzymes. As the platinum-based anticancer drugs cisplatin, carboplatin, and oxaliplatin are an important component of chemotherapy, gold nanoparticles functionalized with a thiolated PEG monolayer capped with a carboxylate group showed significantly better cytotoxicity than oxaliplatin alone [87].

Good antioxidant activity against ROS that reduces oxidative stress locally, which is required for wound healing, is noticed in the case of SeNPs [88]. Moreover, SeNPs are used for nutritional supplementation because of their lower toxicity and ability to gradually release selenium after ingestion [89]. PdNPs, nanosheets, nanoplates, and nanocrystals are used as a carrying cancer drug system [90], but like other NPs such as AuNPs and AgNPs, very few studies have been taken for pharmaceutical applications in the biomedical field [91].

Maybe the most-used NPs in different fields including pharmacy are silver nanoparticles. Especially when functionalized with various groups, AgNPs can be used as adsorbents of metal ions for agricultural and environmental purposes. In pharmacy, they are used as antimicrobial, antifungal, and anti-inflammatory agents [92,93,94].

Among various types of MO NPs, the most used in clinical practice in wound healing dressings or as biosensors or as antimicrobial, anticancer, and image contrast agents are ZnO NPs, Fe_2_O_3_ NPs, Ag_2_O NPs, MgO NPs, TiO_2_ NPs, CeO_2_ NPs, NiO NPs, ZrO_2_ NPs, and CdO NPs [95]. ZnO NPs are a nontoxic, biocompatible biomaterial with strong antibacterial properties and inherent anticancer activity; therefore, ZnO NPs have been approved by the FDA for antitumor therapy.

When using metal-based nanoparticles, the most possible disadvantage and concern comes from researchers not being secure on their impact on health. Due to their small size, it is not completely confirmed how metallic nanoparticles end up in the body and how they affect health in the long term since a number of studies revealed that after inhalation or oral exposure, NPs accumulate in the lungs, digestive tract, liver, heart, spleen, kidneys, and cardiac muscle. For example, the toxicity of TiO_2_ NPs proven by *in vitro* and *in vivo* tests confirmed the toxic effects of TiO_2_ NPs on the human body, such as an altered cell cycle, constriction of nuclear membranes, and apoptosis, as well as DNA damage [96].

The unique features of inorganic and metallic-based nano-structured materials have sparked significant interest in several scientific disciplines, leading to ongoing investigations in this subject. Their applications have already resulted in the creation of innovative and useful products [97]. Nano-structured materials have gained significant attention in recent years due to their distinct physical and chemical properties, as well as their biological properties and functionality resulting from their nano-scale size. These materials have generated great interest and have found important applications in optics and biomedicine [98,99].

A significant element of nanotechnology involves the creation of metal nanoparticles through a synthesis process that is free from any harmful substances. This presents a considerable hurdle. Insights gained from studying nature have resulted in the creation of biomimetic methods for producing sophisticated nano-materials [100].

Due to the rise in antibiotic-resistant microbial organisms and the ongoing focus on healthcare expenses, numerous researchers have endeavored to create novel and efficient antimicrobial drugs that are not susceptible to resistance and are cost-effective. The emergence of these issues and requirements has resulted in a renewed interest in the utilization of nano-sized antiseptics, which are potentially associated with a wide range of effectiveness and a significantly reduced likelihood of causing microbial resistance compared to antibiotics [101].

**Figure 3:**
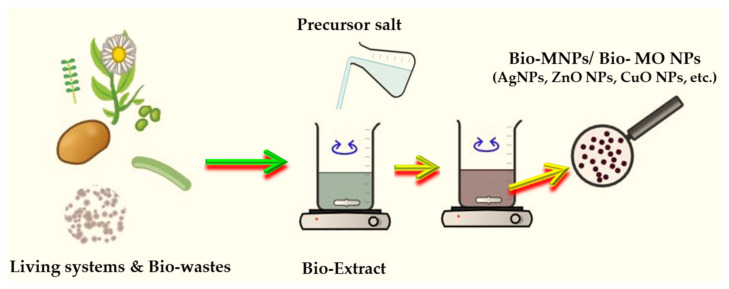
Schematic representation of “green” synthesis of MNPs/MONPs. Figure was created with Chemix (https://chemix.org/, accessed on 21 March 2024) and with PowerPoint and Paint 3D version 1.0.46.0.

MNPs and MONPs can be synthesized by physical and chemical methods, but by electrochemical or photochemical methods as well. Since the physical methods include expensive synthesis and little yield, and the chemical methods are unsafe due to the involvement of hazardous chemical substances that are attached to the surface of MNPs/MONPs, research has been shifted towards the synthesis of MNPs using biological methods that are economical, biocompatible, and non-toxic. This is called the biosynthesis or “green” synthesis. The biosynthesis of MNPs/MONPs has been reported in different microorganisms, including bacteria, actinomycetes, fungi, yeasts, and viruses as well [102]. The “green” synthesis of MNPs/MONPs is schematically displayed in Figure 3.

The plants are the most used as bio-nano-factories for the “green” development of MNPs/ MONPs because they are abundant, safe, and renewable raw materials and contain many valuable bioactive compounds that surround the NPs and impart biological activities to “green” NPs, and they also have biocompatibility, stability, and less or no toxicity.

Nanoparticles can be synthesized through three distinct methods using plants: the first method involves the synthesis of nanoparticles within living plants or through intracellular routes, the second method involves the synthesis of nanoparticles using plant extracts, and the third method involves the synthesis of nanoparticles through the use of phytochemicals [103]. The last two methods include the extracellular pathway for nanoparticle production. The plant contains several primary and secondary metabolites, including proteins, flavonoids, terpenoids, organic acids, alkaloids, and more. These compounds serve as bioreducing and manufacturing agents for the creation of nanoparticles.

The “green” synthesized nanoparticles exhibit many actions, including antibacterial, antifungal, and anticancer properties. As a result of these extensive activities, nanoparticles are recognized for their diverse biological and electronic uses, as well as in the textile industry and also in wastewater treatment. Thus, phyto-synthesized nanoparticles such as CuO NPs, ZnO NPs, MgO NPs, Fe_2_O_3_ NPs, Fe_3_O_4_ NPs, AgNPs, and AuNPs showed excellent photodegradation efficiency toward organic dyes in wastewater [104].

Moreover, as compared to the MNPs/MONPs obtained through classical methods, the phyto-developed MNPs/MONPs possess enhanced bioperformances such as antibacterial, antifungal, antiviral, antioxidant, anti-inflammatory, and anti-mutagenic activities, and were more biocompatible with healthy cells and more harmful to malignant cells. Silver-based NPs phytosynthesized from medicinal plants (*Mentha piperita*, [105]) and weeds (*Andropogon halepensis*, [106]) presented urease inhibitory action. Therefore, these NPs can be used in the biomedical field to develop novel “green” nano-inhibitors of urease, since this enzyme is known as a virulence factor for several microbial pathogens and it is involved in the pathogenesis of many diseases such as gastritis, gastric ulcers, and gastric cancer.

**Figure 4:**
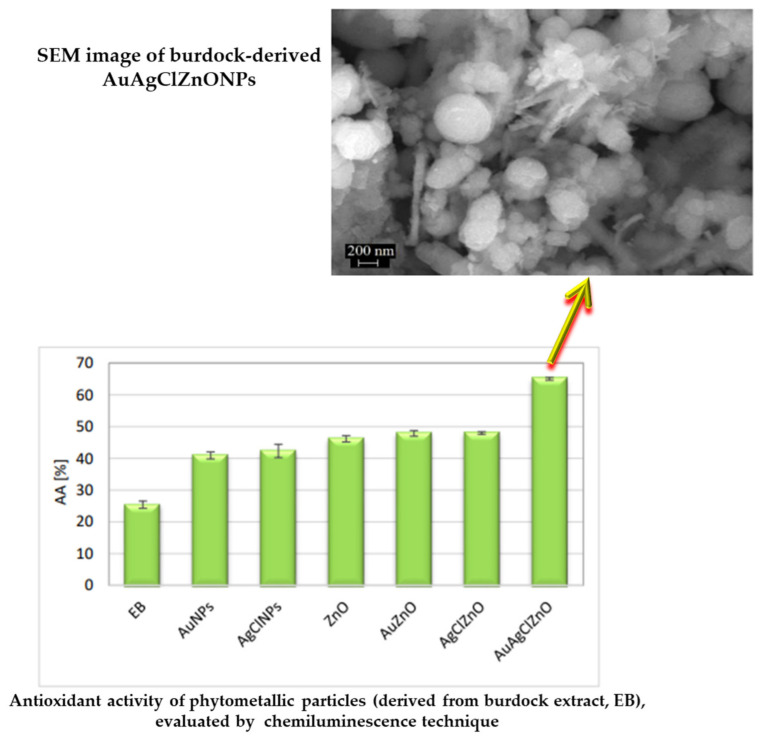
SEM image and the antioxidant activity (AA%) of AuAgClZnONPs phyto-generated from burdock extract (BE) (Adapted upon [107]).

Burdock (*Arctium lappa* L.) aqueous extract was used to generate metal and semiconducting particles (AuNPs, AgClNPs, ZnO, AuZnO, AgClZnO, and AuAgClZnO) with efficient photocatalytic activity, as well as antioxidant and antimicrobial properties. The tricomponent system AuAgClZnO showed the best antioxidant activity for capturing ROS and ABTS•^+^ radicals, and the best antibacterial activity against *Escherichia coli*, *Staphylococcus aureus,* and *Pseudomonas aeruginosa* (see Figure 4). These “green” developed composites can be used as potential adjuvants in the biomedical field (antioxidant or biocidal agents) or in environmental protection (as antimicrobial agents and photocatalysts for the degradation of water pollutants) [107].

Some examples of MNPs/MONPs and their bio-applications including biomedicine, magnetic resonance imaging, magnetic separation and visualization, and hyperthermia of neoplasms are presented in Table 2.

Biohybrids based on phytosynthesized MNPs/MO NPs could be promising “green” platforms with potential bio-pharmaceutical applications to eradicate malignant cells and to eliminate bacteria. Thus, the biomodification, with biomimetic membranes, of the AgNPs generated from an aqueous extract of *Caryophyllus aromaticus* led to the enhancement of bioperformances such as antibacterial activity against *Escherichia coli* and antiproliferative activity against colorectal cancer cells [142]. Biohybrids containing turmeric-generated nano-silver/silver chloride particles, lecithin-liposomes, and CS showed a good free radical scavenging capacity, good antimicrobial activity against *Enterococcus faecalis*, and antiproliferative activity against two cancer cell lines (human colorectal adenocarcinoma cells HT-29 and human liver carcinoma cells HepG2) [143]. The biocomposites AuAgClZnO, phyto-generated from burdock extract, showed good antioxidant activity and biocidal action against *Escherichia coli*, *Staphylococcus aureus*, and *Pseudomonas aeruginosa*. These “green” composites can be applied as adjuvants in biomedical (antioxidant or biocidal agents) or environmental (as antimicrobial agents and catalysts for the degradation of water pollutants) fields [107].

#### 2.6.2. Quantum Dots

**Figure 5:**
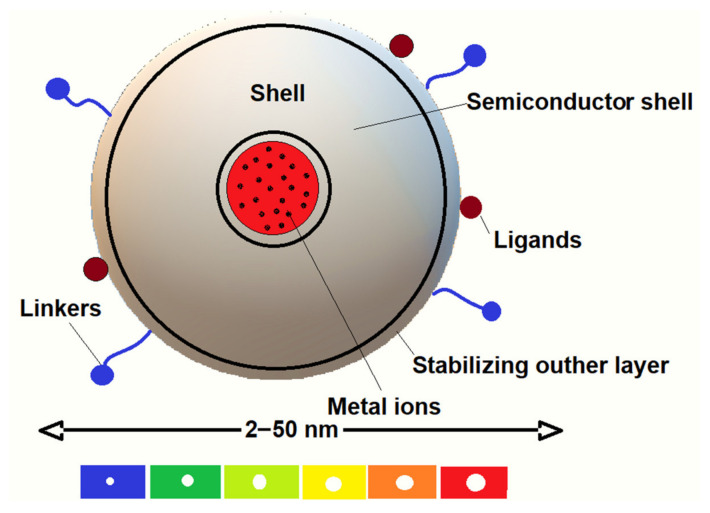
Illustration of a Quantum dot architecture. Figure was created with PowerPoint.

Quantum dots (QDs) are fluorescent colloidal nanocrystals with a diameter between 2 and 10 nm (Figure 5) that consist of an inorganic core responsible for their optical properties and a semiconductor shell, with a lining 1–10 nm in diameter, usually of cadmium selenide (CdSe), cadmium telluride (CdTe), indium phosphide (InP), and indium arsenide (InAs). They can be synthesized by colloidal synthesis or electrochemically. The main use of QDs is in bio-shooting, where these particles serve as contrast agents, giving much better resolution than existing fluorescent colors which allows them use as a diagnostic tool for the *in vitro* and *in vivo* detection and analysis of many biomolecules. Under certain conditions they can become cytotoxic. Also, the most important feature for the application of fluorescent QDs in pharmaceutical sciences is their high surface-to-volume ratio enabling QDs’ conjugation to multiple ligands [144].

Having in mind the potential toxic impact, accumulation, and trophic transfer of QDs, the available knowledge indicates that the development of new Cd-free QDs, for example, would be beneficial for health and the environment [145]. Due to numerous advantageous properties, QDs were also studied in the intracellular transport of D-penicillamine-coated quantum dots (DPA-QDs). Unlike larger nanoparticles, these small DPA-QDs of a 4 nm size were observed to accumulate at the plasma membrane prior to internalization [146].

The coating of QDs with biomolecules, such as DNA, bovine serum albumin, peptides, antibodies, biotin, and folic acid, results in “greener” QDs with a low toxicity and high biocompatibility. Nowadays, “green” methods to prepare “green” QDs have been developed. In this way, the water is used as a “green” solvent. Moreover, the plants and the microorganisms (such as fungi and bacteria) are used for QDs biosynthesis [147].

#### 2.6.3. MOFs (Metal–Organic Frameworks) in Biomedical Applications

Metal–organic frameworks (MOFs) have garnered significant attention in biomedical applications due to their unique properties and versatile structures. These porous materials, composed of metal ions or clusters connected by organic ligands, offer a range of opportunities in the field of medicine [148].

MOFs provide an excellent platform for drug delivery systems. Their high surface area and tunable porosity allow the encapsulation and controlled release of therapeutic agents. This controlled release can enhance the efficacy of drugs while minimizing side effects [149]. Some MOFs exhibit intrinsic antimicrobial properties, making them useful in the development of antimicrobial agents. Additionally, MOFs can be loaded with antimicrobial agents for controlled release and combating infections more effectively [150].

The inherent tunability of MOFs enables the incorporation of imaging agents such as fluorescent dyes or contrast agents for various imaging modalities (e.g., MRI, CT, and fluorescence imaging). This makes MOFs valuable in diagnostics and the monitoring of diseases. The applications of Fe(III)-based MOFs in these three significant fields represent a cohesive approach to innovative strategies [151,152]. MOFs have been explored in the development of biosensors for the detection of biomolecules. Their high surface area and tailored pore structures make them suitable for immobilizing biomolecules, leading to enhanced sensitivity and selectivity in biosensing applications. On the other hand, MOFs characterized by elongated crystalline formations consisting of metal clusters enveloped by organic linkers exhibit significant promise in advancing biosensor development [153].

MOFs can be designed as theranostic platforms, integrating both therapeutic and diagnostic functions. This enables simultaneous imaging and treatment, providing a personalized and targeted approach to medicine [154].

MOFs can be engineered to incorporate photosensitizers for photodynamic therapy. Upon exposure to light, these MOFs generate reactive oxygen species, leading to localized cell death. This makes them promising candidates for cancer therapy [155].

MOFs have become a focal point in biomimetic catalysis research. Nevertheless, precisely adjusting the activity of MOFs by tuning the coordination of metal nodes remains a substantial challenge. Taking inspiration from metalloenzymes with well-defined coordination structures, a series of MOFs containing halogen-coordinated copper nodes (Cu-X MOFs, where X=Cl, Br, I) is utilized to explore their structure–activity relationship. Mechanistically, the antioxidant and antiapoptotic properties of Cu–Cl MOF are achieved by regulating the NRF2 and JNK or P38 MAPK pathways [156].

MOFs have emerged as promising materials in photothermal therapy (PTT) due to their unique properties. PTT is a therapeutic approach that utilizes light-absorbing agents to convert optical energy into heat, selectively targeting and destroying cancer cells. In addition to photothermal therapy, magnetic hyperthermia (MHT) presents a compelling approach to nanoparticle-mediated thermal treatment. Unlike PTT, MHT imposes fewer constraints on tissue penetration through electromagnetic waves, making it effective for treating various solid tumors, including deep-tissue ones [157]. MHT is recognized as a potent adjunct to chemotherapy, demonstrating superior synergistic effects compared to other hyperthermia methods. Notably, MHT has been shown to stimulate the individual’s own antitumor immune responses. Currently, superparamagnetic nanoparticles, such as superparamagnetic iron oxide nanoparticles and superparamagnetic gold nanoparticle clusters, are employed for MHT in tumor treatment due to their excellent magnetic properties and biocompatibility. Consequently, the development of magnetic nanocomposites for synergistic magnetic hyperthermia with chemotherapy is gaining increasing attention, offering a promising avenue to enhance tumor therapeutic efficiency [158].

MOFs have emerged as highly promising materials for CO_2_ reduction due to their exceptional attributes, including an extensive surface area, customizable architectures, pronounced porosity, abundant active sites, and well-distributed metallic nodes. Various materials, specifically MOFs based on nickel, cobalt, zinc, and copper, highlighted their efficacy in facilitating efficient CO_2_ conversion. The unique properties of these MOFs contribute to their effectiveness in catalyzing the electrochemical reduction of CO_2_ [159].

While the potential of MOFs in biomedical applications is promising, challenges such as toxicity, stability, and large-scale production need to be addressed for their successful translation from the laboratory to clinical settings. Ongoing research aims to overcome these challenges and unlock the full potential of MOFs in improving medical treatments and diagnostics.

### 2.7. Bio-Nanopharmaceuticals

Bio-nanopharmaceuticals are a special type of nano-scaled pharmaceuticals prepared from biomolecules (e.g., peptides, proteins, polysaccharides, nucleic acids), entire living systems, or bio-derived entities. A recent review [57] detailed the main types of biomolecular and cell-derived nanocarriers used in biomedical applications. They are biocompatible and biodegradable, and their breakdown products are not harmful. Human cells, plant cells, yeast cells, and diatom cells have been used as drug delivery vehicles in various biomedical applications due to their safety, biocompatibility, and efficient bioencapsulation ability [57].

#### 2.7.1. Red Blood Cells (RBCs)

Among the cell-based drug delivery systems, Red Blood Cells (RBCs) are the most convenient choice, because RBCs have a long lifespan and they are missing all major organelles (e.g., nucleus, mitochondria, etc.) that are targeted by medicinal agents [160].

The hybrid structures based on living cells functionalized with polymers and/or nanoparticles are firstly termed as “cyborg cells” by Fakhrullin et al. [161]. These hybrids combine the intrinsic biological functions of living cells with the functionality of the polymers/nanomaterials. Interestingly, after modification with nanomaterials or with polymers, the cells can remain active or remain in hibernation without being killed [162]. These “cyborg cells” have attractive applications in the biomedical field (biosensing, cell-therapy, delivery, tissue regeneration, etc.). For example, exoskeleton structures of MOF nanoparticles were developed by metal–phenolic coordination for RBCs encapsulation. In these cyborg hybrids, RBCs’ membranes and MOF NPs were complexed through hydrogen bonding, without the lysis of RBCs [162]. Such hybrids possess resistance against harsh factors and can be used for multimodal imaging and for sensing the blood’s nitric oxide.

The derived structures from living cells are also used to develop new drug delivery vehicles. Thus, RBCs’ membrane-derived nanoparticles have emerged as a promising platform for drug delivery due to their unique properties, including biocompatibility, stability, and ability to evade immune recognition [163]. These nanoparticles, derived from the membranes of RBCs, offer numerous advantages for therapeutic applications, ranging from targeted drug delivery to the treatment of various diseases. This review explores the diverse applications of RBC membrane-derived nanoparticles and discusses the key challenges hindering their clinical translation. RBC membrane-derived nanoparticles hold significant potential in biomedical applications, particularly drug delivery and disease treatment [164,165]. The lack of a nucleus and organelles in RBCs makes their membranes easy to process, allowing for the encapsulation of therapeutic payloads within the nanoparticle core. These nanoparticles can be functionalized with targeting ligands to enhance specificity towards diseased tissues or cells, thereby improving drug efficacy and minimizing off-target effects. Furthermore, RBC membrane-derived nanoparticles exhibit inherent stealth properties, enabling prolonged circulation times in the bloodstream and reduced clearance by the immune system. This stealth capability is attributed to surface proteins and glycans that mimic those found on native RBCs, allowing the nanoparticles to evade phagocytic clearance and systemic immune responses [166]. The versatility of RBC membrane-derived nanoparticles extends beyond drug delivery, encompassing applications in imaging, diagnostics, and regenerative medicine. By leveraging RBC membranes’ natural biocompatibility and biodegradability, researchers have developed multifunctional nanoparticles capable of delivering contrast agents for imaging modalities, such as magnetic resonance imaging and fluorescence imaging [167].

Additionally, RBC membrane-derived nanoparticles have been explored for their potential in targeted therapy against cancer, inflammatory disorders, and infectious diseases. The ability to modulate the surface chemistry of these nanoparticles through conjugation with specific ligands allows for the precise targeting of pathological tissues while minimizing adverse effects on healthy cells. Despite the promising preclinical results and vast potential of RBC membrane-derived nanoparticles, several challenges must be addressed for their successful clinical translation. One of the primary challenges is scalability and reproducibility in nanoparticle manufacturing [167,168]. The production of RBC membrane-derived nanoparticles at an industrial scale remains complex and costly, requiring optimized purification methods and standardized protocols to ensure batch-to-batch consistency. Moreover, concerns regarding RBC membrane-derived nanoparticles’ long-term safety and immunogenicity must be thoroughly investigated. While the biomimetic nature of these nanoparticles confers biocompatibility, potential immunogenic responses to foreign proteins and antigens in the RBC membrane components must be evaluated through comprehensive preclinical studies and immunotoxicity assessments. Another critical aspect that hinders clinical translation is the regulatory approval process for nanoparticle-based therapeutics [168]. The regulatory landscape for novel drug delivery systems, including RBC membrane-derived nanoparticles, constantly evolves, requiring stringent preclinical testing and regulatory submissions to ensure safety, efficacy, and compliance with regulatory standards [169,170,171]. Furthermore, the clinical development of RBC membrane-derived nanoparticles requires interdisciplinary collaboration between researchers, clinicians, regulatory agencies, and industry partners. Collaborative efforts are essential for advancing nanoparticle-based therapies from bench to bedside, encompassing preclinical studies, clinical trials, and post-market surveillance to assess long-term safety and efficacy outcomes [164].

Further, we discuss a particular type of bionanopharmaceuticals, namely Prebiotics, Probiotics, and Postbiotics.

#### 2.7.2. Prebiotics, Probiotics, and Postbiotics in Nanopharmaceuticals

The microbiome within the intestines forms a highly intricate ecological community. Recognizing that its overall quality plays a crucial role in influencing both physical and mental health, it is worthwhile to familiarize oneself with its essential “residents”—probiotics, prebiotics, and postbiotics. They may represent the next generation of medicines [172,173].

*Probiotics*

An altered microbiota can be partially restored and affected through probiotic supplementation [174]. Probiotics exhibit significant potential in treating and preventing a range of diseases, including neurodegenerative disorders, cancers, cardiovascular diseases, and inflammatory conditions. They have a multitude of positive effects on our body, and here are just some of them:

- Maintaining a healthy microflora of the digestive system.
- Regulating digestion.
- Exhibiting anti-carcinogenic activity.
- Reducing cholesterol levels in the blood.
- Stimulating the immune system.

However, the clinical application of probiotics faces challenges due to their status as living microorganisms. Biological and biopharmaceutical barriers, such as susceptibility to harsh gastric conditions and bile salts, limit their efficacy [175]. Conditions in the digestive tract pose challenges to the viability of probiotics during *in vivo* transportation. The encapsulation technologies emerge as a promising solution to address this issue. There is lack of information about the probiotic effect after encapsulation on its antibacterial and antioxidant activity. Traditional encapsulation methods face limitations such as susceptibility to extreme temperatures, larger capsule sizes, and difficulties in controlling particle sizes. The evolution from traditional techniques to innovative approaches, including bulk encapsulation using nanofibers and nanoparticles, as well as the application of nanobiofilms, biological membranes, and nanocoating for individual probiotics, can improve probiotic biopharmaceutical barriers [176,177].

Double emulsion microbial encapsulation is a promising way to provide probiotic living cells protected from environmental conditions. The probiotics’ protection is dependent on emulsification methods, emulsifier selection, the effect of probiotics, and the modification of emulsification techniques, as well as the targeted release mechanisms [178]. Polysaccharide-encapsulated probiotics are encapsulated by technologies including extrusion, emulsion, spray-drying, freeze-drying, and electrohydrodynamics [179,180].

Biopolymers such as alginate and gelatin are also used as probiotic carriers. The encapsulation of *Propionibacterium freudenreichii* in alginate–gelatin capsules participates in delivering probiotic bacteria to the intestine [181]. An alginate hydrogel microsphere encapsulating *Bifidobacterium* (Bac) and drug-modified nanoscale dietary fibers (NDFs) is responsible for protecting drugs from acidic and multi-enzymatic environments and delivering drugs to the colorectum [182]. Also, interestingly, similar studies have shown that utilizing biopolymer-based edible films containing probiotics is a promising approach to preserving minimally processed fruits and vegetables (MPFVs). Their ability to safeguard the viability of probiotics is essential for ensuring the overall effectiveness of MPFVs [183]. Some authors suggest that probiotics can also be isolated from fermented food products of animals and plants. A novel category, the so-called next-generation probiotics (NGPs), of recently isolated microorganisms has great potential of health benefits [184]. The effects of probiotics in several conditions and their way of action are mainly related to the production of short-chain fatty acids (SCFA) [185].

The effectiveness of oral medications in treating ulcerative colitis (UC) has been limited by low drug accumulation in the colitis mucosa, leading to suboptimal therapeutic outcomes. A novel high-performance nanotherapeutic has been developed, consisting of a pluronic F127 (P127)-modified gold shell (AuS) encapsulating a polymeric core loaded with curcumin (CUR) [186].

To further enhance the therapeutic effect, nanoprobiotics, the probiotic-derived outer membrane vesicles (OMVs)—encapsulating manganese dioxide nanozymes—have been constructed, which can adhere to an inflamed colonic epithelium and eliminate intestinal excess reactive oxygen species. Insignificant systemic toxicity in this treatment was observed [187]. Probiotics like artificial-enzyme-modified *Bifidobacterium longum* probiotics play a crucial role in enhancing the targeting and retention of biocompatible artificial enzymes, enabling them to effectively and persistently scavenge the elevated ROS level and alleviate inflammatory factors [188].

An *E. coli*-derived membrane (EM) as the surface and the biodegradable diselenide-bridged mesoporous silica nanoparticles (SeM) as the core restored the intestinal redox balance and immune regulation homeostasis in a murine model of acute colitis induced by dextran sodium sulfate [189].

Kefiran, an exopolysaccharide derived from the microflora of kefir, has garnered significant attention from researchers due to its diverse biological properties, biocompatibility, and versatile applications, ranging from nanomedicine to food packaging. The versatility of kefiran in various forms, from composites to nanofibers, coupled with its diverse applications, makes it a promising material with significant potential in the realms of food technology and medical sciences. Recent advancements demonstrate that combining kefiran with other polymers such as whey protein isolate and waterborne polyurethane, along with the incorporation of essential oils (EO) and nanofillers like nanocellulose, zinc oxide, and alumina nanoparticles, results in nanocomposites with barrier and mechanical properties comparable or superior to synthetic polymers commonly used in standard packaging materials [190].

*Prebiotics*

Unlike probiotics, prebiotics are not living organisms, they are food for probiotics. These are ballast substances, such as inulin or galactooligosaccharides, which cannot be digested in the small intestine and so come to the large intestine, where they serve as nutrients for good bacteria. Therefore, prebiotics can be used as an additional support for probiotics. Prebiotics mainly consist of complex carbohydrates and fibers found in a large number of different plant foods [191]. Prebiotics can be recognized by the following names: galactooligosaccharides, fructooligosaccharides, oligofructose, inulin, and chicory fiber. By consuming plant-based products, most of the fiber they contain passes through your stomach and small intestine relatively intact because humans lack the enzymes to break it down. But, microbes in the colon can metabolize fiber and break it down into other compounds [192].

Prebiotics emerge as optimal ingredients for nano-encapsulation and oral drug delivery, capitalizing on their inherent capacity to shield the encapsulated compounds during transit through the upper gastrointestinal (GI) tract [193].

*Postbiotics*

Instead, postbiotics are created during the digestive process after microbe fibers break down. Compounds produced by one type of bacteria can be the food (or prebiotic) that another type of bacteria depends on. A group of postbiotic compounds called *short-chain fatty acids (SCFAs)* are extremely good for the health. One of the best-studied SCFAs is butyrate. This compound helps maintain gut health by serving as a fuel source for the cells lining your colon. Butyrate helps reduce inflammation and mediates the immune system. It affects brain health and can stimulate the production of Glucagon-like peptide 1 (GLP-1), a hormone that suppresses appetite.

Numerous *in vivo* and *in vitro* experiments have consistently demonstrated that extracellular NPs produced by beneficial microbiota play a crucial role in conferring distinctive health-promoting functions. These effects extend beyond the intestinal locale to encompass systemic impacts, thereby introducing a novel concept known as *postbiotics* [194,195].

The effect of *L. casei* postbiotics (LCP) at a sub-minimum inhibitory concentration on the expression of QS genes, including lasR/I, rhlR/I, pqsA, pqsR, and virulence genes including pelF (pellicle/biofilm glycosyltransferase PelF), lasB (elastase LasB), and toxA (exotoxin A) reducing the virulence and biofilm development of *P. aeruginosa*, suggested a novel safe natural source for the expansion of anti-virulence treatments [196].

The poor availability of scientific research on nanotechnology concerning probiotics, postbiotics, and prebiotics implies dynamic research studies on the bioavailability of loaded active ingredients and the effective drug delivery system are needed.

**Figure 6:**
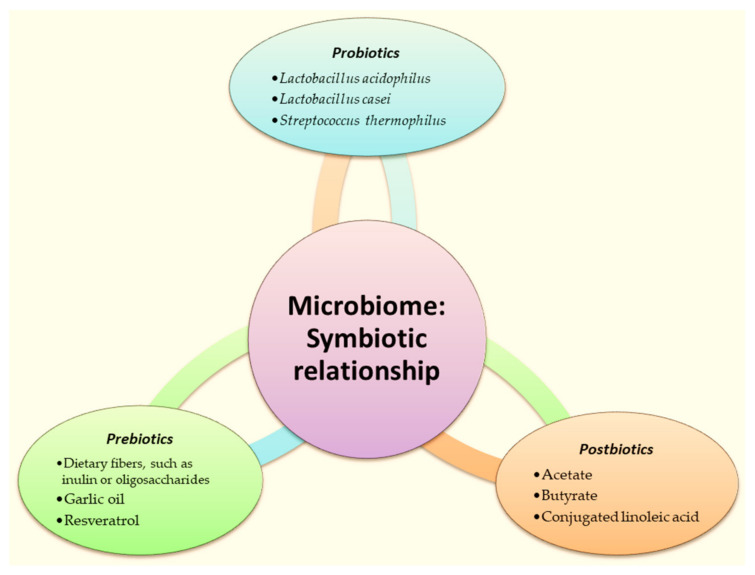
Schematic representation of symbiotic relationship in microbiome. Figure was created Microsoft PowerPoint.

A schematic representation of the symbiotic relationship in the microbiome is illustrated in Figure 6.

## 3. Current Trends in Modern Nanopharmaceuticals´ Design

The current trends in modern nanopharmaceuticals´ design are nanosuspension technology, nano-encapsulation, 3D printing, biomimetics and bioinspiration, and green design.

### 3.1. Nanosuspension Technology

Nanosuspension technology is an attractive nanotechnological approach to improve the pharmaceutical potential of drugs and plant extracts by enhancing their solubility and their oral bioavailability. The nanoprecipitation method proved successful in formulating a nanosuspension. The nanoprecipitation approach was used for the preparation of a plant-derived nanosuspension by dissolving the plant extract in an organic phase (ethanol) and then filtering. The resulting solution was gradually added, under constant stirring, into an aqueous phase containing a stabilizer which can be a surfactant or a polymer. Pharmaceutical-grade nanosuspensions with a minimal particle size and polydispersity index were achieved by Zafar et al. from *Allium cepa* peel extract [197] and from *Terminalia arjuna* bark extract [198], and stabilized with sodium lauryl sulfate and polysorbate-80, respectively. The formulated nanosuspensions were found physically stable and non-toxic, and exhibited bioactivities.

### 3.2. Nano-Encapsulation

*Nanocapsules* have emerged as promising for delivering pharmaceuticals, nutraceuticals, and other bioactive compounds. Their unique structure, consisting of a core–shell architecture with a diameter typically ranging from 10 to 1000 nm [199], allows for precise control over drug release kinetics, stability, and targeting. This review explores the diverse materials and formulation techniques employed in fabricating nanocapsules, highlighting their potential applications and future directions in drug delivery and beyond [199,200].

Materials for Nanocapsule Formulation:

*Nanocapsules* can be fabricated from various materials, offering distinct advantages and characteristics. Common materials include polymers, lipids, and inorganic nanoparticles. Nanocapsules are used as drug delivery systems for multiple medications through diverse routes of administration, such as oral and parenteral, for reducing drug toxicity, and enhancing drug stability [201]. Nanocapsules are considered active vectors due to their ability to release medications, and their small size enables better cellular targeting.

**Figure 7:**
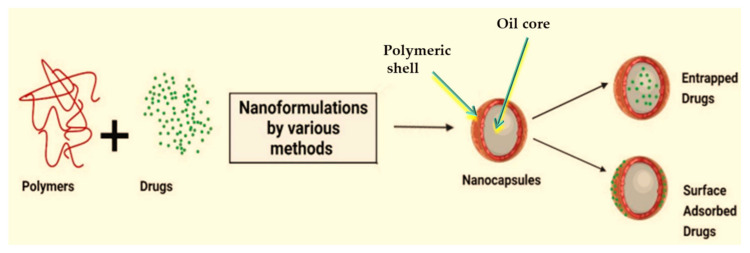
Schematic representation of the structure of nanocapsules (adapted upon [202]).

*Polymeric Nanocapsules:* polymeric nanocapsules may contain an oleic core that is ideal for enclosing lipophilic substances, and a polymeric shell that can control the release profile of the drug (Figure 7) [202].

Polymers such as poly(lactic-co-glycolic acid) (PLGA), poly(lactic acid) (PLA) [201], and CS are widely used in the formulation of nanocapsules due to their biocompatibility, biodegradability, and tunable properties. These polymers can be synthesized via emulsion polymerization, nanoprecipitation, and self-assembly, allowing for precise control over particle size, morphology, and drug loading [201]. Shell materials are crucial in the creation of polymeric nanocapsules for the storage, safeguarding, and releasing of bioactive compounds. The polymers’ characteristics significantly affect the stability, encapsulation efficiency, release profile, and biodistribution of the nanocapsules used as drug delivery systems [201]. Biocompatible polymeric materials are widely regarded as suitable options for developing nanocapsules. Typically, these polymers must be biodegradable to release the payload and remove nanoparticles. Non-biodegradable but biocompatible polymers like PEG and polyvinyl alcohol (PVA) are commonly utilized in creating nanoparticles. They can help in medication release by diffusion due to their hydrophilic nature. Furthermore, they can be eliminated from the bloodstream through the reticuloendothelial system, even if not broken down into smaller molecules [203,204]. Various polymers are used to create nanocapsule shells to meet diverse application needs. These polymers can be categorized as natural or synthetic based on their origin. Polysaccharides, a crucial type of natural polymeric material, are commonly utilized as drug carriers due to their biocompatibility, gelation conditions, and mucoadhesive qualities. Polysaccharides typically contain deprotonated amino or carboxylic acid groups, which can have cationic or anionic charges. This forms a polymeric shell through electrostatic attractive interactions [203]. Chitosan (CS), a typical natural polymer, is widely utilized as a drug carrier due to its biocompatibility, ability to be metabolized naturally, gelation capabilities, and ability to adhere to mucous membranes. Nanocapsules coated with CS can develop a positive surface charge due to the many amino groups in CS [204]. The positively charged surface can enhance the interaction between nanoparticles and bacteria with negatively charged surfaces through electrostatic interactions.

**Figure 8:**
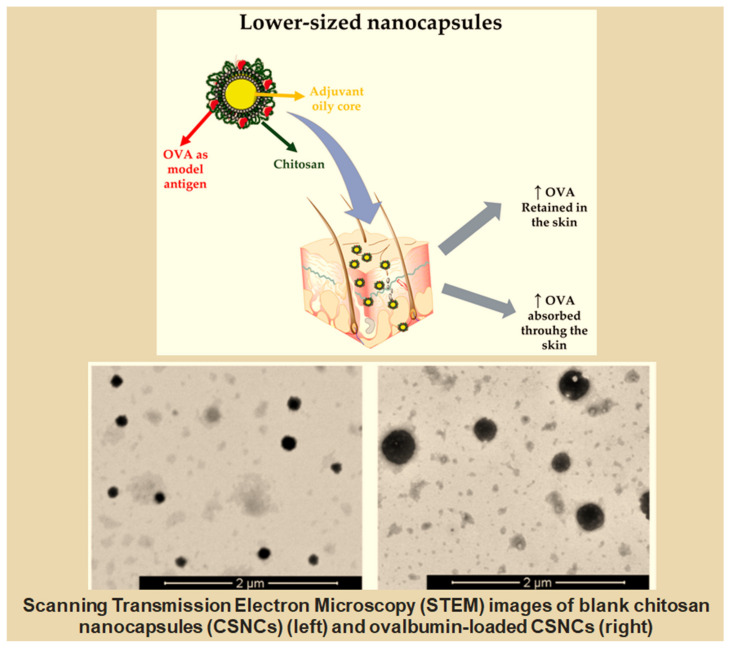
Lower-Sized Chitosan Nanocapsules for Transcutaneous Antigen Delivery (adapted from [205]).

Bussio et al. designed and developed chitosan nanocapsules (CSNCs) with a spherical shape, small size, and a positive zeta potential. The obtained CSNCs promoted the transdermal penetration of ovalbumin (OVA, used as the antigen model) and exhibited increased retention in the skin (Figure 8). Thus, this carrier can be an excellent platform for transcutaneous antigen delivery [205].

PLGA nanocapsules with CS shells showed improved adhesion to *S. aureus* and *M. abscessus* compared to those without CS [203,204]. CS-based nanocapsules have been created as a medicine delivery device for infectious diseases [204]. Yet, the potent cationic surface charge can lead to nanoparticle aggregation, protein adsorption, and rapid elimination from the bloodstream. Various anionic polymers, including PAA, PVA, and anionic polysaccharides, have been utilized alongside CS to serve as nanocapsule shells to address this issue [206].

Alginate, an anionic natural polysaccharide, is used in drug carrier nanoformulations due to its biocompatibility, minimal immunogenicity, and gentle gelation conditions. Aside from its established benefits, alginate is a pH-responsive polymer that can shield payloads in acidic settings and release drugs in alkaline environments. Nanocapsules made from alginate have been created as a possible method for delivering drugs to the intestines by oral administration. Dextran sulfate, a biocompatible and biodegradable polyanionic polymer, is commonly used in the pharmaceutical industry for drug delivery. Dextran sulfate and CS are typically combined to create multilayer nanocapsules using electrostatic integration [204]. Nanocapsules composed of CS and dextran sulfate exhibited excellent stability without requiring additional covalent agents. The drug release behavior can be altered by adjusting the ratio of CS to dextran. Chitosan–dextran nanoparticles with more carboxymethyl dextran can enhance nanoparticle dissociation, increasing the gene release in serum or the cytoplasm. In addition to the common polysaccharides, poly(cyclodextrin), heparin, hyaluronan, and other polysaccharides have been utilized to create nanocapsules for drug delivery in various pharmaceutical applications [204,206,207]. Due to their biocompatibility and adjustable features, protein-based polymers are used as polymeric shells for nanocapsules. Albumin is a water-soluble and biodegradable protein that plays a crucial role in the circulatory system [207]. Human serum albumin has been used as a casing for nanocapsules. The albumin corona not only regulates the drug penetration rate, but also decreases the immunogenicity of nanoparticles, helping them evade the reticuloendothelial system’s reconfiguration. Albumin’s biogenic qualities make it a suitable targeting ligand for albondin receptors, which are highly expressed on endothelial cells of tumor blood arteries, creating an effective medication targeting mechanism. Protein can be designed to form a hollow-caged nanostructure by self-assembling a specific number of subunits [206]. The virus-like biomimetic nanocapsules offer a precise size distribution and function as a drug delivery method. A HspG41C mutant protein-based nanocapsule, constructed by self-assembling 24 monomeric proteins, was created as a carrier for the anti-cancer medication doxorubicin. The HspG41C nanocapsule can be readily created through a self-assembly process in a water environment, resulting in particles approximately 12 nm in size with a uniform size distribution. The material exhibited excellent biocompatibility and effectively transported doxorubicin to several cancer cell types [207]—a protein-based caged nanostructure for delivering doxorubicin as a drug delivery method. The structure was designed by self-assembling 60 dihydrolipolyl acyltransferase subunits (E2), resulting in a particle size of around 25 nm [208].

Synthesized materials offer advantages over natural materials due to their consistent quality and purity. Furthermore, they can be customized with chemical ionic, mechanical, solubility, and degradability properties to suit various pharmaceutical uses. Aliphatic polyesters and related copolymers are widely used synthetic polymers that are extensively researched and utilized for drug delivery systems because of their biocompatibility and biodegradability. Standard polyesters include poly (lactic acid) (PLA) [207,208], poly (lactic-co-glycolic acid) (PLGA), and poly(ε-caprolactone) (PCL). PCLs offer a significantly longer degradation period compared to PLA and PLGA copolymers. Thus, PCLs are more suited for long-term drug delivery systems or medicinal applications. Furthermore, some research indicates that PCLs are more cost-effective than PLAs and PLGAs [209].

*Lipid-based nanocapsules:* Lipid-based nanocapsules, including liposomes, SLNs, and NLCs, offer unique advantages such as a high drug-loading capacity, stability, and controlled release kinetics [57,210]. Lipids such as phospholipids, triglycerides, and cholesterol are commonly used to formulate lipid-based nanocapsules, which can be prepared via solvent evaporation, lipid film hydration, and microemulsion.

*Inorganic nanocapsules:* Inorganic nanoparticles, such as mesoporous silica nanoparticles (MSNPs), gold nanoparticles (AuNPs), and magnetic nanoparticles, provide additional functionalities such as controlled release, targeting, and imaging [207]. These nanoparticles can be synthesized via bottom-up approaches such as sol–gel chemistry, template-directed synthesis, and chemical vapor deposition, allowing precise control over the size, shape, and surface properties.

Vegetable oils, including soybean oil and palm oil, together with fatty acids and medium-chain triglycerides, are optimal selections for the oily core of nanocapsules due to their capacity to break down lipophilic medications and the safety they offer to the oil phase. The oil serves as a medicine solvent and provides therapeutic advantages, making it a good option for the core of nanocapsules [211,212].

Copaiba oil was used as the core material in a PCL nanocapsule to improve the solubility of imiquimod, a hydrophobic anti-cancer drug. Copaiba oil has therapeutic effects for malignant melanoma and micropapillary carcinoma and possesses anti-inflammatory and analgesic qualities. Copaiba oil not only serves as a core for drug encapsulation, but also demonstrates anti-inflammatory and anti-proliferative effects that aid in therapy [213].

Turmeric and lemongrass oils are used as oil cores due to their antibacterial, antifungal, antioxidant, antimutagenic, and anticarcinogenic properties. Polymeric nanocapsules can be created with a water-based core to act as a base for the sustained release of hydrophilic substances. Gemcitabine hydrochloride and doxorubicin, hydrophilic anti-cancer drugs, have been efficiently encapsulated in polymeric nanocapsules containing an aqueous core. Nanocapsules containing drugs showed better effectiveness in fighting cancer than the medicine alone [212]. Polymeric nanocapsules containing an aqueous core can efficiently enclose and safeguard hydrophilic bioactive compounds. The clinical application of mono- or oligo-nucleotides is limited due to their low stability and ability to pass through cell membranes in biological environments. Encapsulating polymeric nanocapsules in the aqueous core can protect them against degradation in biological fluids and increase their capacity to enter cells, thus enhancing their availability [214]. Polymeric nanocapsules containing an aqueous core can effectively distribute water-soluble proteins such as albumin. Furthermore, nanocapsules can have a hollow internal structure. Nanocapsules with a hollow core are often produced by creating a solid sphere, which is extracted after the polymeric shell is formed [215]. A solid sphere as a sacrificial template can provide a sturdy spherical framework for creating nanocapsules with many polymer layers. Eliminating the solid sphere template to form a hollow core is crucial for regulating medication release within a living organism and improving the compatibility of the nanocapsules. The materials identified as the template core should be easily removed under mild conditions to avoid shell damage [216]. Calcium carbonate, an inorganic core template for nanocapsules, can be removed by adding ethylene diamine tetraacetic acid to an aqueous dispersion of nanocapsules at pH 7 [217]. Silica is frequently used as a template core material and it can fully dissolve in hydrofluoric acid. Polystyrene can be converted into nanospheres using nanoemulsion and then processed with trichloromethane to generate a cavity, which is later eliminated [218].

*Nanocapsules* represent a versatile platform for delivering bioactive compounds, offering precise control over drug release kinetics, stability, and targeting. By leveraging a diverse array of materials and formulation techniques, researchers can tailor the properties of nanocapsules to suit a wide range of applications in drug delivery, diagnostics, and therapeutics. Continuing research into novel materials, formulation techniques, and fabrication methods will drive further advancements in nanocapsule-based drug delivery, paving the way for improved treatments and therapies for various diseases and disorders.

### 3.3. Three-Dimensional Printing in Nanopharmacy (NanoPrinting)

Integrating 3D printing technology into the realm of nanopharmaceuticals heralds a paradigm shift in drug development and delivery. This comprehensive review delves into the transformative potential of 3D printing, exploring its applications in fabricating intricate nanostructures and personalized drug formulations. From its inception to its current prominence, 3D printing has emerged as a disruptive force, offering unparalleled precision and versatility in creating nanoscale drug carriers and medical devices [219]. Through a detailed examination of the synthesis methodologies, materials, and applications, this review elucidates the evolving landscape of 3D printing in nanopharmaceutical development, paving the way for a future where precision medicine meets advanced manufacturing. We delve into the rationale behind integrating 3D printing into drug development, highlighting its potential to revolutionize drug delivery systems and personalized medicine. This chapter sets the stage for a deeper exploration into the synthesis methodologies, materials, and applications of 3D printing in nanopharmaceuticals [220,221].

#### 3.3.1. Fundamentals of 3D Printing

In the fundamental principles of 3D printing, to elucidate the various techniques and processes employed in fabricating nanoscale structures [221], from stereolithography (SLA) and selective laser sintering (SLS) to extrusion-based printing, each method is dissected to uncover its unique advantages and limitations [222]. The discussion extends to the materials used in 3D printing and exploring their suitability for creating drug carriers with precise control over size, shape, and composition. Three-dimensional printing technology has revolutionized various industries, including healthcare and pharmaceuticals. In nanopharmaceutical development, 3D printing offers unprecedented precision and versatility in fabricating intricate nanostructures for drug delivery and personalized medicine. This section delves into the fundamental principles of 3D printing, elucidating the various techniques, processes, and materials employed in creating nanoscale drug carriers and medical devices [223]. At its core, 3D printing, also known as additive manufacturing, involves the layer-by-layer deposition of material to create three-dimensional objects based on digital designs [224]. This additive process contrasts with subtractive manufacturing methods, such as milling or machining, where the material is removed from a solid block [225]. The versatility of 3D printing lies in its ability to fabricate complex geometries with high precision and customization. Several techniques are employed in 3D printing, each offering unique advantages and limitations [225,226,227]. Among the most common techniques used in nanopharmaceutical development are stereolithography, selective laser sintering, and extrusion-based printing. Stereolithography utilizes a photosensitive resin that solidifies when exposed to light, allowing for the creation of detailed structures layer by layer. Selective laser sintering involves fusing powdered materials using a high-powered laser, while extrusion-based printing deposits material through a nozzle to build up layers [227]. The process of 3D printing typically begins with the creation of a digital model using computer-aided design (CAD) software—AutoCAD 2018 software (Autodesk Inc., San Rafael, CA, USA). This model is then sliced into thin horizontal layers, and the printing process begins layer by layer [228]. Depending on the technique to create the desired object, the material may be deposited, solidified, or fused. Post-processing steps, such as curing or surface finishing, may enhance the final product’s properties [229]. Many materials can be used in 3D printing, including polymers, metals, ceramics, and composites. In nanopharmaceutical development, biocompatible and biodegradable polymers are often preferred for fabricating drug carriers and medical devices [230]. Common polymers used include PLA, PLGA, and PEG. These materials offer excellent mechanical properties, biocompatibility, and the ability to degrade in the body over time. The applications of 3D printing in nanopharmaceutical development are vast and diverse [231]. From fabricating drug-loaded nanoparticles and microparticles for controlled drug delivery to creating patient-specific implants and medical devices [232], 3D printing offers unparalleled customization and precision. The ability to tailor drug formulations to individual patient needs and fabricate complex structures with precise control over the size, shape, and composition has revolutionized drug development and personalized medicine [232,233]. The fundamentals of 3D printing are foundational to understanding its applications in nanopharmaceutical development. By mastering the principles, techniques, processes, and materials of 3D printing, researchers and pharmaceutical scientists can harness its transformative potential to revolutionize drug delivery systems, personalized medicine, and patient care. As technology advances, the future of 3D printing in nanopharmaceuticals holds promise for further innovation and breakthroughs in healthcare [234].

#### 3.3.2. Synthesis of Nanopharmaceuticals *via* 3D Printing

This section navigates the synthesis methodologies for fabricating nanopharmaceuticals using 3D printing technology. We explore how 3D printing enables the creation of complex drug delivery systems, including nanoparticles, liposomes, and micelles, with tailored properties for enhanced drug release kinetics and targeting [235]. Case studies highlight the versatility of 3D printing in fabricating personalized drug formulations tailored to individual patient needs. As the demand for personalized medicine and targeted drug delivery grows, innovative technologies are needed to meet these evolving healthcare needs. Among these technologies [232], 3D printing has emerged as a promising tool for synthesizing nanopharmaceuticals with precise control over the size, shape, and composition [232,235]. This section explores the synthesis methodologies employed in fabricating nanopharmaceuticals *via* 3D printing, highlighting the diverse range of applications and the transformative impact on drug delivery systems [236]. The synthesis of nanopharmaceuticals *via* 3D printing encompasses a variety of fabrication techniques, each tailored to specific applications and materials. Among these techniques, stereolithography stands out for its ability to create intricate nanostructures with high resolution and accuracy [237]. Selective laser sintering offers versatility in working with a wide range of materials. At the same time, extrusion-based printing enables the deposition of drug-loaded polymers with precise control over drug release kinetics [238]. The selection of materials plays a critical role in synthesizing nanopharmaceuticals *via* 3D printing. Biocompatible and biodegradable polymers, such as PLGA and PEG, are commonly used for fabricating drug carriers and medical devices [238,239]. In addition to polymers, nanoparticles and nanocomposites can be incorporated into the printing process to impart specific functionalities, such as targeted drug delivery or enhanced therapeutic efficacy. One of the key advantages of 3D printing in nanopharmaceutical synthesis is the ability to encapsulate drugs within the printed structures with precise control over drug loading and release kinetics. By incorporating drugs into the printing process, researchers can tailor drug formulations to achieve desired release profiles, ranging from sustained release to pulsatile or on-demand release. This flexibility opens up new possibilities for designing personalized drug delivery systems tailored to individual patient needs [240,241].

Surface functionalization and modification are essential aspects of nanopharmaceutical synthesis *via* 3D printing, enabling the incorporation of targeting ligands, imaging agents, or stimuli-responsive moieties into the printed structures [242]. Through surface engineering, researchers can enhance the specificity and efficacy of drug delivery systems, enabling targeted delivery to diseased tissues while minimizing off-target effects. Surface modification techniques such as click chemistry, layer-by-layer assembly, and covalent conjugation offer precise control over the attachment of functional groups to the printed nanocarriers [243]. Characterization and optimization are critical in synthesizing nanopharmaceuticals *via* 3D printing, ensuring the printed formulations’ reproducibility, stability, and efficacy. Physicochemical characterization techniques, including scanning electron microscopy (SEM), dynamic light scattering (DLS), and Fourier-transform infrared spectroscopy (FTIR) [244], provide insights into the structural properties and drug release behavior of the printed nanocarriers. Optimization strategies, such as the design of experiments (DoE) and mathematical modeling, enable researchers to fine-tune printing parameters and formulation compositions to achieve the desired performance metrics. The synthesis of nanopharmaceuticals *via* 3D printing represents a transformative approach to drug delivery systems, offering unprecedented precision, customization, and control over drug release kinetics [245]. By leveraging advanced fabrication techniques, material selection, drug encapsulation strategies, and surface modification techniques, researchers can design next-generation nanopharmaceuticals tailored to address specific disease conditions and patient needs [246]. As technology advances, the future of nanopharmaceutical synthesis *via* 3D printing holds promise for revolutionizing personalized medicine and improving patient outcomes [247].

#### 3.3.3. Applications in Drug Delivery Systems

The versatility of 3D printing in drug delivery systems takes center stage as we delve into its applications in formulating oral, transdermal, and injectable dosage forms. The discussion spans controlled release formulations, mucoadhesive drug carriers, and patient-specific implants, showcasing how 3D printing technology revolutionizes drug delivery by offering precise control over drug release profiles and site-specific targeting [248]. Researchers have turned to 3D printing as a versatile tool for fabricating nanopharmaceuticals. This section explores the diverse applications of 3D printing in drug delivery systems, highlighting its ability to tailor drug formulations, control drug release kinetics, and enhance therapeutic efficacy. From oral and transdermal dosage forms to implantable devices and targeted drug carriers, 3D printing offers unparalleled versatility and precision in drug delivery. One of the most promising applications of 3D printing in drug delivery systems is fabricating oral dosage forms with tailored drug release profiles [248,249]. By incorporating drugs into biocompatible polymers and controlling the printing parameters, researchers can design oral tablets, capsules, and multiarticulate formulations with precise control over drug release kinetics. These formulations enable sustained release, pulsatile release, or site-specific targeting, enhancing drug efficacy and patient compliance [250]. Transdermal drug delivery systems offer a non-invasive route for drug administration, bypassing the gastrointestinal tract and avoiding first-pass metabolism. Three-dimensional printing allows for fabricating transdermal patches and films with customized drug loading and release properties. By encapsulating drugs within biodegradable polymers or hydrogels, researchers can design transdermal formulations that provide a sustained release over extended periods, offering continuous therapeutic effects and improved patient comfort. Injectable drug delivery systems are crucial in delivering therapeutics directly into the bloodstream or target tissues, bypassing the gastrointestinal tract or skin barriers [249]. With 3D printing, researchers can fabricate injectable microparticles, nanoparticles, and hydrogels with precise control over the size, shape, and drug release kinetics. These formulations enable targeted delivery to specific sites of action, minimizing systemic side effects and enhancing therapeutic efficacy [251].

Implantable drug delivery devices offer a promising approach for sustained and localized drug delivery, particularly for chronic diseases or conditions requiring long-term treatment regimens. Three-dimensional printing enables the fabrication of implantable devices with intricate geometries and tailored drug release profiles [248,249,250,251]. By incorporating drugs into biocompatible materials such as polymers or ceramics, researchers can design implants that release therapeutics over extended periods, providing continuous and controlled drug delivery directly to the target site. Targeted drug delivery systems aim to deliver therapeutics selectively to diseased tissues while minimizing exposure to healthy tissues, thereby reducing systemic side effects and improving therapeutic outcomes. Three-dimensional printing allows for the fabrication of targeted drug carriers, such as nanoparticles, liposomes, and micelles, with precise control over the size, shape, and surface functionalization. By conjugating targeting ligands or antibodies onto the surface of these carriers, researchers can achieve site-specific targeting, enhancing drug accumulation and efficacy at the diseased site [252].

The applications of 3D printing in drug delivery systems are vast and diverse, offering unprecedented opportunities for personalized and targeted therapeutics. Three-dimensional printing enables precise control over drug release kinetics, formulation compositions, and drug targeting from oral and transdermal dosage forms to implantable devices and targeted drug carriers. As researchers continue to explore the potential of 3D printing in nanopharmaceuticals, the future holds promise for innovative drug delivery solutions that improve patient outcomes and quality of life [251,252].

#### 3.3.4. Personalized Medicine and Patient-Specific Formulations

Transitioning seamlessly into personalized medicine, this section explores how 3D printing enables the fabrication of patient-specific drug formulations tailored to individual genetic, physiological, and clinical parameters [248,249,250]. The discussion encompasses personalized implants, prosthetics, and medical devices, illustrating how 3D printing technology empowers clinicians to deliver optimized treatment regimens with unprecedented precision. Personalized medicine has gained significant traction as a paradigm shift in healthcare, aiming to tailor medical treatments to individual patient characteristics, needs, and preferences. Central to the realization of personalized medicine is the development of patient-specific formulations in drug delivery systems, which enable precise dosing, targeted delivery, and optimized therapeutic outcomes. This section explores the evolving landscape of personalized medicine and the pivotal role of patient-specific formulations in drug delivery [251].

Personalized medicine represents a departure from the traditional one-size-fits-all approach to medical treatment, recognizing that individual patients exhibit unique genetic, physiological, and environmental factors that influence their response to therapy. By leveraging advances in genomics, proteomics, and other omics technologies, healthcare practitioners can identify biomarkers and molecular signatures that inform treatment decisions and guide the selection of tailored therapies [252]. Personalized medicine holds promise for optimizing drug efficacy, minimizing adverse reactions, and improving patient outcomes across various diseases and conditions. Central to the success of personalized medicine is the development of drug delivery systems that accommodate the diverse needs and characteristics of individual patients. Patient-specific formulations offer several advantages over conventional dosage forms, including precise dosing, targeted delivery to specific tissues or cell types, and optimized drug release kinetics. By tailoring drug formulations to individual patient parameters such as age, weight, genetics, disease stage, and comorbidities, healthcare practitioners can optimize therapeutic efficacy while minimizing the risk of adverse effects [252].

Recent advancements in drug delivery technologies have paved the way for developing patient-specific formulations with enhanced precision and customization. Nanotechnology-based drug delivery systems, such as nanoparticles, liposomes, and micelles, offer versatile platforms for encapsulating and delivering therapeutics with precise control over drug release kinetics and targeting specificity [246,252]. Three-dimensional printing technologies enable the fabrication of personalized dosage forms with customized drug loading, release profiles, and geometries, allowing for tailored treatment regimens based on individual patient needs. Precision oncology represents a prominent application of personalized medicine, leveraging molecular profiling to guide the selection of targeted therapies and immunotherapies tailored to cancer patients’ unique genetic mutations and biomarker profiles. Patient-specific formulations play a crucial role in precision oncology by delivering anticancer agents directly to tumor cells while sparing healthy tissues, maximizing therapeutic efficacy and minimizing systemic toxicity. Nanoparticle-based drug delivery systems enable the encapsulation of chemotherapeutic agents, targeted drugs, and immunotherapies for precise delivery to tumor sites, enhancing treatment outcomes and the patients’ quality of life [253].

While personalized medicine holds immense promise for revolutionizing healthcare, several challenges remain to be addressed in developing and implementing patient-specific formulations. These include regulatory hurdles, technological limitations, cost considerations, and ethical implications of data privacy and informed consent. Nevertheless, continued advancements in drug delivery technologies, biomarker discovery, and computational modeling hold the potential to overcome these challenges and usher in a new era of precision medicine tailored to the needs of individual patients [251,252,253].

Personalized medicine represents a transformative approach to healthcare, leveraging patient-specific formulations in drug delivery systems to optimize therapeutic outcomes and improve patients’ quality of life. By tailoring drug treatments to individual patient characteristics and disease profiles, personalized medicine is promising to enhance treatment efficacy, minimize adverse effects, and advance the frontier of precision oncology and other therapeutic areas. As researchers and clinicians continue to innovate in the field of drug delivery, the future of personalized medicine shines bright with possibilities for improving patient care and shaping the future of healthcare [254].

**Figure 9:**
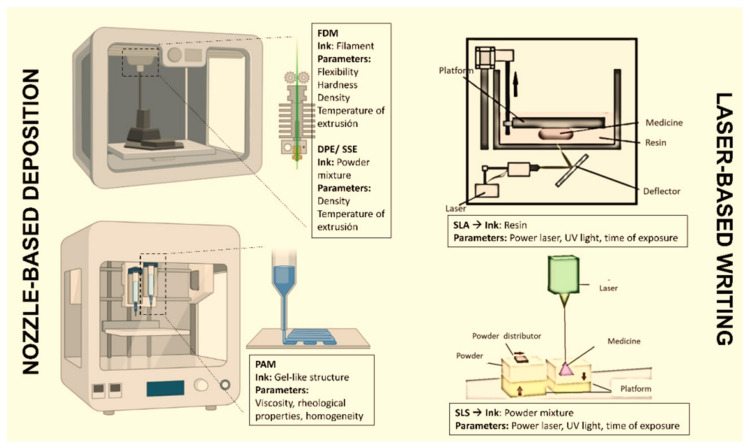
The 3D printing techniques commonly used in the fabrication of personalized medicines (abbreviations: fuse deposition modeling, FDM; direct powder extrusion, DPE; semisolid extrusion, SSE; pressure-assisted microsyringes, PAM; stereolithography, SLA; selective laser sintering, SLS) [255].

The 3D printing techniques commonly used in the development of personalized medicines are displayed in Figure 9 [255].

**Figure 10:**
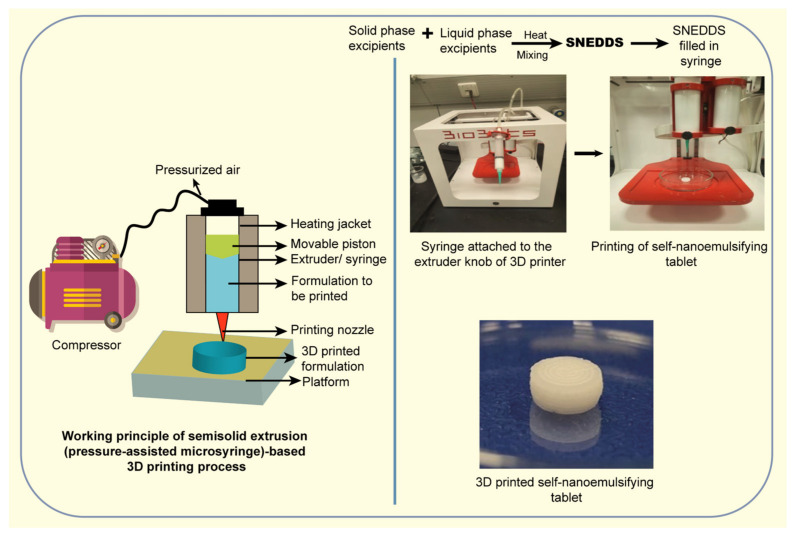
The workflow of 3D printing of dapagliflozin-containing self-nanoemulsifying tablets [256].

An interesting study reported the preparation of self-nanoemulsifying drug delivery systems (SNEDDS) in the form of 3D-printed tablets encapsulating dapagliflozin, an antidiabetic drug [256]. The combination of SNEDDS and the 3D printing technology approach provides an alternative to obtain self-nanoemulsifying solid dosage forms for poorly water-soluble drugs, and the ability to dispense tailored doses for diabetic patients (Figure 10).

### 3.4. Biomimetics and Bioinspiration in Nanopharmaceuticals/Nanomedicines

Nanotechnology has revolutionized various fields, including medicine, by offering unprecedented opportunities to design and develop innovative therapeutic approaches. Biomimetics and bioinspiration have emerged as powerful paradigms in nanopharmaceuticals and nanomedicines, drawing inspiration from nature’s intricate designs and processes. This novel text explores the intersection of nanotechnology with biomimetics and bioinspiration, elucidating how these synergistic approaches are reshaping the landscape of drug delivery, diagnostics, and therapeutics at the nanoscale [257].

Biomimetics, also known as biomimicry, is a field of study that draws inspiration from biological systems, processes, and structures to develop innovative solutions for engineering, technology, and design challenges [258,259]. By emulating the principles and strategies found in nature, biomimetics seeks to create sustainable, efficient, and adaptive technologies that can address complex problems across various disciplines. One of the key aspects of biomimetics is its interdisciplinary nature, involving collaboration between biologists, engineers, physicists, chemists, and designers. Researchers can better understand and replicate living organisms’ remarkable adaptations and functionalities by integrating knowledge from different fields [259].

In medicine and biotechnology, biomimetics has led to advancements such as bio-inspired drug delivery systems, tissue-engineered scaffolds, and artificial organs designed to mimic natural tissues’ and organs’ structures and functions [260]. Researchers aim to develop more effective and biocompatible treatments for various diseases and injuries by incorporating biological principles into medical technologies. Overall, biomimetics offers a promising approach to innovation by leveraging the wealth of solutions already present in the natural world. By learning from nature’s designs, biomimetic researchers aim to create more sustainable and resilient technologies that address pressing global challenges while minimizing the environmental impact [261].

Biomimetics in nanopharmaceuticals involves emulating biological structures, processes, and systems to design advanced nanoscale drug delivery platforms and therapeutic approaches. By mimicking the intricate designs and functions found in nature, biomimetic nanopharmaceuticals offer innovative solutions to address challenges in drug delivery, including enhancing targeting efficiency, improving biocompatibility, and optimizing drug release kinetics [262].

One key aspect of biomimetics in nanopharmaceuticals is the development of nanocarriers that replicate the properties of biological entities such as cells, viruses, or extracellular vesicles [263]. These biomimetic nanocarriers can leverage natural mechanisms, such as receptor-mediated endocytosis or cell adhesion, to enhance the delivery of therapeutic agents to specific target sites within the body [264]. For example, liposomes, which are spherical vesicles composed of lipid bilayers, can mimic cell membranes and facilitate the targeted delivery of drugs to specific cells or tissues. Furthermore, biomimetic nanopharmaceuticals can be engineered to replicate biomolecules’ structural and functional characteristics, such as proteins or nucleic acids, for targeted drug delivery and controlled release [264,265]. For instance, nanoparticles coated with cell-penetrating peptides or protein ligands can mimic the interactions between biological molecules and cell receptors, enabling the efficient intracellular delivery of therapeutic payloads. Another area of biomimetics in nanopharmaceuticals involves the development of nanomaterials with properties inspired by those found in natural systems [266]. For example, nanoparticles coated with biomimetic polymers or proteins can mimic the stealth properties of biological membranes, allowing for prolonged circulation in the bloodstream and reduced immune recognition [267]. Additionally, biomimetic nanoparticles can be designed to respond to external stimuli, such as changes in pH or temperature, similar to how biological systems regulate their functions in response to environmental cues [268]. Moreover, biomimetics in nanopharmaceuticals can be applied to overcome biological barriers, such as the blood–brain barrier or mucosal surfaces, by designing nanocarriers that mimic the transport mechanisms and interactions observed in biological systems [269]. These biomimetic nanocarriers promise to improve the therapeutic delivery to target sites that are otherwise difficult to reach using conventional drug delivery approaches. Overall, biomimetics in nanopharmaceuticals offers a versatile and multidisciplinary approach to drug delivery, leveraging insights from biology, material science, and nanotechnology to develop novel therapeutic strategies with enhanced efficacy and safety profiles [270]. By harnessing the complexity and efficiency of natural systems, biomimetic nanopharmaceuticals have the potential to revolutionize the diagnosis and treatment of various diseases [270,271].

Biomimetics in nanomedicines represents an innovative approach that draws inspiration from biological systems to design nanoscale drug delivery systems and therapeutic interventions [272]. By mimicking biological entities’ structures, functions, and behaviors, biomimetic nanomedicines offer novel strategies to overcome biological barriers, enhance targeting efficiency, and improve therapeutic outcomes [273]. One area of biomimetics in nanomedicines focuses on developing nanocarriers that replicate the properties of biological membranes, such as lipids or proteins [274]. These biomimetic nanocarriers can mimic cell membranes’ dynamic nature and selective permeability, allowing for the efficient encapsulation and delivery of therapeutic agents [275]. By leveraging lipids’ self-assembly properties or membrane-bound proteins’ specificity, biomimetic nanocarriers can enhance drug solubility, stability, and bioavailability. Furthermore, biomimetic nanomedicines can be designed to target specific cells or tissues by incorporating targeting ligands or receptors that recognize and bind to molecular markers expressed on diseased cells [276]. For example, nanoparticles coated with antibodies or aptamers can selectively target cancer cells or inflamed tissues, minimizing off-target effects and improving therapeutic efficacy. Additionally, biomimetic nanoparticles can exploit natural cellular uptake mechanisms, such as receptor-mediated endocytosis, to facilitate intracellular drug delivery and enhance therapeutic payload delivery to the desired site of action [276,277]. Another aspect of biomimetics in nanomedicines involves the development of nanomaterials with properties inspired by those found in biological systems [278]. For instance, nanoparticles coated with biomimetic polymers or stealth coatings can evade immune recognition and prolong circulation time in the bloodstream, enhancing drug delivery efficiency and reducing systemic toxicity [279]. Moreover, biomimetic nanomedicines can be engineered to respond to specific biological stimuli, such as changes in pH or temperature, allowing for controlled drug release at target sites [280]. Also, biomimetic nanomedicines can replicate biological tissues or organs’ complex structures and functions for applications to regenerative medicine. By mimicking the extracellular matrix or cellular microenvironment, biomimetic scaffolds or hydrogels can promote tissue regeneration and repair, offering potential solutions for wound healing, tissue engineering, and organ transplantation [281]. Overall, biomimetics in nanomedicines holds great promise for advancing drug delivery and therapeutic interventions by harnessing the biological systems’ inherent complexity and efficiency. By integrating principles from biology, nanotechnology, and material science, biomimetic nanomedicines have the potential to revolutionize the diagnosis, treatment, and prevention of various diseases, ultimately improving patient outcomes and quality of life [282,283].

Bioinspiration, or biologically inspired design, is the process of deriving ideas, concepts, and solutions from the natural world to address engineering, design, and technological challenges. Unlike biomimicry, which directly replicates biological structures or processes, bioinspiration seeks to abstract and adapt principles from nature for application in various human-made systems [284]. Engineers and designers can develop innovative designs and technologies that exhibit similar functionality and performance by studying the solutions to complex problems and understanding the underlying principles [285].

Bioinspired design often involves identifying analogies between natural systems and human-made problems and then translating these analogies into practical solutions. This process requires interdisciplinary collaboration between biologists, engineers, designers, and other experts to bridge the gap between biological knowledge and technological innovation. Bioinspiration offers a powerful approach to innovation by leveraging nature’s ingenuity to solve human challenges. Drawing inspiration from the natural world, researchers and designers can create more sustainable, efficient, and resilient technologies that benefit society and the environment. Bioinspiration in nanopharmaceuticals harnesses the principles observed in biological systems to design and develop innovative drug delivery platforms and therapeutic strategies at the nanoscale. By drawing inspiration from nature, researchers aim to overcome various challenges in drug delivery, such as improving targeting specificity, enhancing therapeutic efficacy, and reducing side effects [286].

One key area of bioinspiration in nanopharmaceuticals is the design of nanocarriers that mimic biological entities such as cells or viruses. These biomimetic nanocarriers can exploit natural processes, such as cellular uptake mechanisms or virus–host interactions, to enhance drug delivery to specific target tissues or cells [286]. For example, liposomes, which are spherical vesicles composed of lipid bilayers, can mimic cell membranes and facilitate the targeted delivery of drugs to cells via receptor-mediated endocytosis. Another aspect of bioinspiration in nanopharmaceuticals involves the development of nanomaterials with properties inspired by those found in biological systems. For instance, researchers have designed nanoparticles with surface modifications that mimic the stealth properties of red blood cells, enabling prolonged circulation in the bloodstream and improved immune system evasion [287]. Furthermore, bioinspired nanopharmaceuticals can be designed to replicate the functionality of natural biological structures, such as extracellular vesicles or exosomes, which play crucial roles in intercellular communication and molecular transport. These biomimetic nanocarriers promise to deliver therapeutic payloads, including nucleic acids and proteins, to target cells with high precision and efficiency. Moreover, bioinspiration in nanopharmaceuticals extends to developing drug delivery systems inspired by biological barriers and transport mechanisms. For example, researchers have engineered nanoparticles coated with biomimetic peptides or proteins to facilitate transport across biological barriers, such as the blood–brain barrier, to deliver therapeutics to the central nervous system [288].

Overall, bioinspiration in nanopharmaceuticals offers a multidisciplinary approach to drug delivery and therapy development, leveraging the sophistication and efficiency of biological systems to design next-generation nanomedicines with an enhanced therapeutic performance and reduced toxicity. By integrating principles from biology, material science, and nanotechnology, bioinspired nanopharmaceuticals hold great promise for revolutionizing the diagnosis and treatment of various diseases. Bioinspiration in nanomedicines harnesses nature’s ingenuity to develop innovative solutions for diagnosing, treating, and preventing diseases at the nanoscale. By emulating biological structures, processes, and mechanisms, bioinspired nanomedicines offer unique advantages such as improved targeting, enhanced biocompatibility, and reduced side effects. Here, we explore critical aspects of bioinspiration in nanomedicines and its potential applications in healthcare [271].

#### 3.4.1. Targeted Drug Delivery

**Figure 11:**
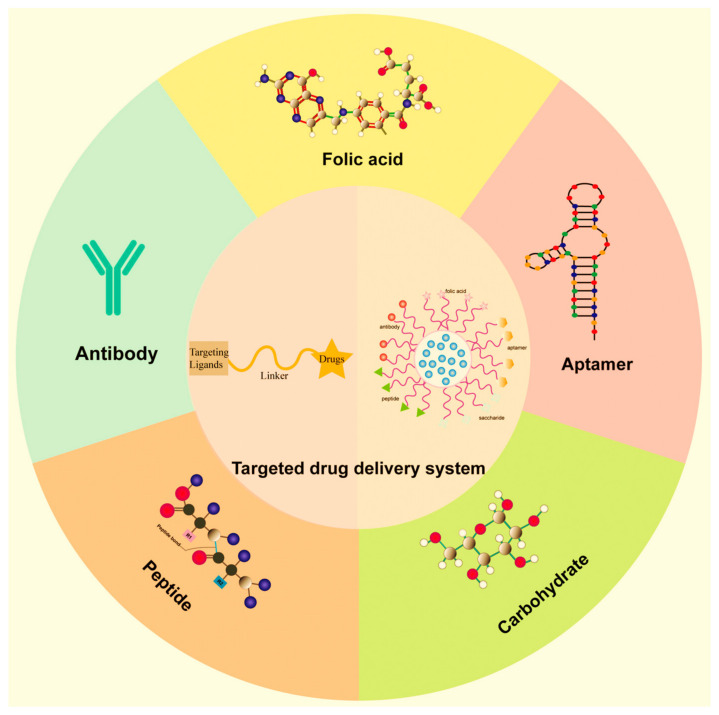
Different targeting ligands used in targeted drug delivery [289].

Nature provides elegant solutions for the targeted delivery of therapeutic payloads to specific cells or tissues. Bioinspired nanomedicines leverage this concept by incorporating targeting ligands (Figure 11), such as antibodies or peptides, that recognize and bind to molecular markers overexpressed on diseased cells [289].

By mimicking the specificity of biological recognition processes, these nanocarriers can selectively deliver drugs to the site of action, minimizing off-target effects and maximizing therapeutic efficacy. Bioinspiration in nanomedicines has revolutionized targeted drug delivery by drawing inspiration from nature’s precision targeting mechanisms. Mimicking biological recognition processes, bioinspired nanocarriers offer enhanced specificity and efficacy in delivering therapeutic payloads to diseased cells or tissues while minimizing off-target effects. Here, we delve into the principles and applications of bioinspired targeted drug delivery in nanomedicine:

1. **Biological Targeting Ligands:** Bioinspired nanomedicines utilize targeting ligands, such as antibodies, peptides, or aptamers, that recognize and bind to specific molecular markers overexpressed on the surface of diseased cells. By mimicking the specificity of biological recognition processes, these nanocarriers can selectively deliver drugs to the site of action, sparing healthy tissues and minimizing systemic toxicity [290].
2. **Cellular Membrane Camouflage:** Biomimetic nanocarriers can cloak themselves with cell-membrane-derived vesicles, mimicking the surface properties of host cells. This camouflage allows the nanocarriers to evade immune recognition and clearance while promoting interactions with target cells. These bioinspired nanomedicines enhance targeted drug delivery efficiency across biological barriers by leveraging endogenous cellular uptake mechanisms [291].
3. **Exosome-Mimetic Nanovesicles:** Exosomes, natural extracellular vesicles secreted by cells, are carriers for intercellular communication and cargo transport. Bioinspired nanomedicines replicate exosomes’ structural and functional properties to achieve targeted drug delivery. Engineered nanovesicles resembling exosomes can exploit endocytic pathways and cell-to-cell interactions, facilitating specific drug delivery to recipient cells or tissues [292].
4. **Biological Barriers’ Penetration:** Nature-inspired nanomedicines are designed to navigate biological barriers, such as the blood–brain barrier (BBB) or tumor microenvironment, to reach their intended targets. By mimicking biological entities’ size, shape, and surface properties, these nanocarriers can traverse cellular membranes and penetrate tissue barriers more efficiently, enabling effective drug delivery to otherwise inaccessible sites [293].
5. **Stimuli-Responsive Targeting:** Bioinspired nanomedicines incorporate stimuli-responsive elements that enable a triggered drug release in response to specific biological cues. For example, pH-sensitive nanocarriers can selectively release their cargo in acidic microenvironments, exploiting the tumor’s acidic pH to trigger drug release. Similarly, enzyme-responsive nanocarriers can respond to enzymatic activity characteristics of certain diseases, allowing precise control over drug delivery kinetics [264].

Bioinspiration has transformed targeted drug delivery in nanomedicines by harnessing the sophistication of biological systems. By mimicking nature’s precision targeting mechanisms, bioinspired nanocarriers offer unparalleled specificity, efficiency, and safety in delivering therapeutics to diseased tissues. As research advances, bioinspired nanomedicines are promising to revolutionize drug delivery strategies and improve patient outcomes in various medical applications.

#### 3.4.2. Biological Barriers’ Penetration

Biological barriers, such as the BBB or cellular membranes, pose challenges for drug delivery. Bioinspired nanomedicines are designed to navigate these barriers by mimicking the properties of natural carriers. For example, nanoparticles coated with cell membrane-derived vesicles or peptides can exploit endogenous transport mechanisms to penetrate cellular barriers and deliver therapeutics to intracellular targets.

Bioinspiration in nanomedicines has paved the way for innovative strategies to overcome biological barriers, enabling the more effective delivery of therapeutic agents to target sites within the body. Mimicking natural mechanisms observed in biological systems, bioinspired nanomedicines offer promising solutions for penetrating various barriers encountered during drug delivery. Here, we explore how bioinspiration facilitates the penetration of biological barriers in nanomedicine applications:

1. **Cellular Membrane Interactions:** Biological membranes serve as formidable barriers that regulate the entry of molecules into cells. Bioinspired nanomedicines leverage interactions with cellular membranes to facilitate the uptake and intracellular delivery of therapeutic payloads. By mimicking the surface properties and recognition mechanisms of biological entities, such as viruses or extracellular vesicles, nanocarriers can enhance cellular internalization and trafficking to target organelles or cellular compartments [294].
2. **Endocytic Pathways’ Exploitation:** Cells employ endocytic pathways to internalize extracellular materials, including nanoparticles, through clathrin-mediated endocytosis, caveolae-mediated endocytosis, or macropinocytosis—bioinspired nanomedicines design strategies to exploit these endocytic mechanisms for efficient cellular uptake and intracellular drug delivery. By mimicking the endogenous cargoes’ size, shape, and surface properties, such as exosomes or viral particles, nanocarriers can hijack specific endocytic pathways to traverse cellular membranes and access the intracellular space [295].
3. **Blood–Brain Barrier (BBB) Penetration:** The BBB presents a formidable obstacle to drug delivery to the central nervous system (CNS), restricting the passage of therapeutics into the brain parenchyma. Bioinspired nanomedicines mimic the properties of endogenous molecules that traverse the BBB, such as specific peptides or transport proteins, to facilitate brain-targeted drug delivery. Additionally, engineered nanocarriers can exploit receptor-mediated transcytosis or bypass mechanisms to penetrate the BBB while maintaining therapeutic efficacy and minimizing neurotoxicity [296].
4. **Tumor Microenvironment Permeation:** The complex tumor microenvironment poses significant challenges for drug delivery, including heterogeneous blood vessel distribution, elevated interstitial fluid pressure, and dense extracellular matrix components. Bioinspired nanomedicines are designed to navigate these obstacles by mimicking the behavior of leukocytes or other cells that extravasate into tumors. Strategies such as surface modification with cell-adhesion molecules or matrix-degrading enzymes enable nanocarriers to penetrate tumor tissues, enhancing drug accumulation and distribution within the tumor microenvironment [297].
5. **Mucus Barrier Overcoming:** Mucus layers lining mucosal surfaces, such as the respiratory, gastrointestinal, and reproductive tracts, act as protective barriers that limit the penetration of foreign particles, including nanoparticles. Bioinspired nanomedicines draw inspiration from mucolytic enzymes or mucoadhesive molecules found in nature to overcome mucus barriers. Nanocarriers can efficiently penetrate mucus layers by incorporating mucin-binding ligands or enzymatic degradation motifs, enabling targeted drug delivery to underlying tissues or cells [298].

Bioinspiration offers valuable insights for designing nanomedicines capable of penetrating biological barriers encountered during drug delivery. Bioinspired nanocarriers hold tremendous potential for enhancing therapeutic interventions’ efficiency, specificity, and safety in various disease contexts by mimicking the sophisticated strategies employed by biological systems. Continued research in this field promises to unlock new opportunities to address unmet clinical needs and improve patient outcomes in nanomedicine applications.

#### 3.4.3. Stealth and Biocompatibility

Nature-inspired nanomedicines prioritize biocompatibility and stealthiness to evade immune recognition and prolong circulation time in the bloodstream. By mimicking the surface properties of biological entities, such as red blood cells or cell membranes, these nanocarriers can reduce clearance by the immune system and enhance drug delivery efficiency. Biomimetic coatings or stealth polymers enable the prolonged circulation and controlled release of therapeutic agents, improving pharmacokinetics and reducing toxicity. Bioinspiration in nanomedicines has revolutionized the development of stealth nanocarriers with enhanced biocompatibility, aiming to evade the immune system and improve therapeutic efficacy. Drawing inspiration from nature, researchers have devised ingenious strategies to mimic the stealth and biocompatibility mechanisms observed in biological systems. Here, we explore how bioinspired approaches have been employed to enhance the stealth and biocompatibility of nanomedicines:

1. **Stealth Coating Mimicry:** Many biological entities, such as red blood cells and specific pathogens, possess surface coatings that enable them to evade detection by the immune system. Inspired by these natural stealth mechanisms, researchers have developed stealth coatings for nanomedicines to prolong circulation time and reduce immune recognition. For example, PEG has been widely used as a biomimetic stealth coating because it forms a hydrated layer on nanoparticle surfaces, preventing opsonization and clearance by phagocytic cells [299].
2. **Cell Membrane Camouflage:** Biomimetic nanocarriers can be engineered to mimic the surface properties of host cells, such as erythrocytes or leukocytes, through cell membrane coating strategies. By coating synthetic nanoparticles with cell membranes extracted from host cells, nanocarriers acquire a “self” identity that reduces immune recognition and enhances biocompatibility. Cell-membrane-camouflaged nanomedicines evade immune surveillance, exhibit prolonged circulation, and have improved targeting capabilities owing to their biological origin and surface composition [299]. RBC membrane-derived nanoparticles represent a promising platform for drug delivery and biomedical applications. Their inherent biocompatibility, stealth properties, and ability to encapsulate therapeutic payloads make them attractive candidates for targeted therapy against various diseases. However, scalability, safety, and regulatory approval pose significant hurdles in their clinical translation. Addressing these challenges through collaborative research efforts and rigorous preclinical testing is essential for harnessing the full potential of RBC membrane-derived nanoparticles in clinical practice.
3. **Biodegradable Materials Inspiration:** Natural biomaterials with inherent biocompatibility and biodegradability, such as lipids, polysaccharides, and proteins, serve as inspiration for designing nanomedicines with a minimal toxicity and environmental impact. Bioinspired nanocarriers based on biodegradable polymers or lipid formulations mimic biological molecules’ structural and functional properties, ensuring compatibility with physiological systems and facilitating safe degradation and clearance from the body [300].
4. **Surface Modification with Natural Ligands:** Nature provides a wealth of molecular motifs, such as glycoproteins and glycolipids, that mediate specific interactions with biological receptors and signaling pathways. Bioinspired nanomedicines leverage these natural ligands to modify nanoparticle surfaces, enabling targeted delivery and enhanced biocompatibility. By functionalizing nanocarriers with biomimetic ligands, such as carbohydrates or peptide sequences, researchers can achieve selective binding to cell surface receptors or extracellular matrix components, facilitating site-specific accumulation and uptake while minimizing off-target effects [299,300].
5. **Immunomodulatory Biomimetic Agents:** Biological systems employ various immunomodulatory agents, such as cytokines and peptides, to regulate immune responses and maintain homeostasis. Bioinspired nanomedicines integrate immunomodulatory biomolecules into their design to modulate immune reactions and mitigate adverse immune responses. By mimicking the activity of endogenous immunoregulatory factors, such as anti-inflammatory cytokines or immunosuppressive peptides, nanocarriers can promote immune tolerance and enhance biocompatibility *in vivo* [300].

Bioinspired strategies play a pivotal role in enhancing the stealth and biocompatibility of nanomedicines, offering innovative solutions to overcome immune barriers and improve therapeutic outcomes. By harnessing the principles of biomimicry, researchers continue to advance the development of nanocarriers with unprecedented biocompatibility, paving the way for safer and more effective biomedical applications in drug delivery and precision medicine.

#### 3.4.4. Responsive Drug Release

**Figure 12:**
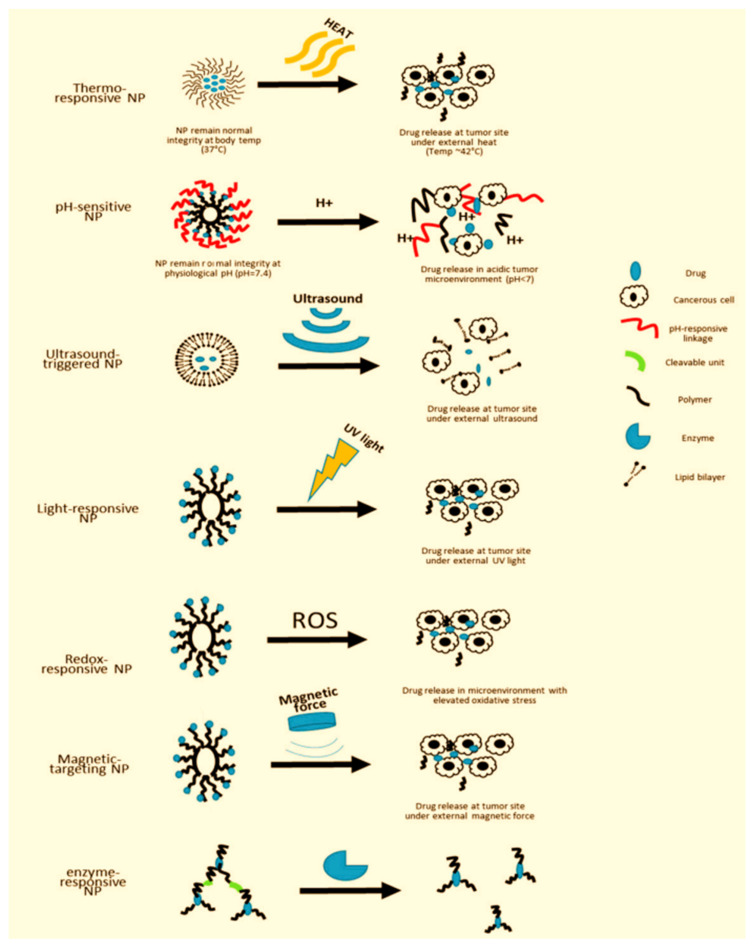
Schematic representation of the drug-releasing mechanisms of different types of drug nanocarriers [301].

Various stimuli, such as changes in pH, temperature, or enzyme activity, the presence of ROS, and also the action of light, ultrasounds, and magnetic force, can trigger drug release from nanocarriers at the desired site of action. Figure 12 illustrates the drug-releasing mechanisms of different types of stimuli-responsive nanoparticles (srNPs) [301].

Bioinspired nanomedicines incorporate responsive elements, such as intelligent polymers or molecular switches, that mimic the dynamic responsiveness of biological systems. These stimuli-responsive nanocarriers enable spatiotemporal control over drug release, enhancing therapeutic efficacy while minimizing systemic side effects. Bioinspiration in nanomedicines has revolutionized the field of responsive drug release systems, enabling precise control over drug delivery in response to specific physiological cues or external stimuli. Drawing inspiration from natural systems, researchers have developed innovative nanocarriers capable of modulating drug release kinetics spatiotemporally controlled. Here, we explore how bioinspired approaches have been leveraged to design nanomedicines with responsive drug release capabilities:

1. **pH-Responsive Nanocarriers:** Biological environments, such as the acidic tumor microenvironment and the acidic compartments within cells, inspire pH-responsive drug delivery systems. Biomimetic nanocarriers engineered to respond to changes in pH exhibit altered physicochemical properties, such as protonation/deprotonation or structural transformations, leading to triggered drug release. By incorporating pH-sensitive moieties, such as acidic or basic functional groups, into nanocarrier formulations, researchers can exploit pH gradients to achieve site-specific drug delivery and enhance therapeutic efficacy while minimizing off-target effects [302].
2. **Enzyme-Responsive Nanoparticles:** Biological systems utilize enzymes for many physiological processes, making them attractive targets for bioinspired drug delivery systems. Nanocarriers designed to respond to specific enzymatic activities can selectively release their cargo in the presence of disease-associated enzymes, such as proteases or nucleases. Researchers can achieve triggered drug release at disease sites by incorporating enzyme-cleavable linkers or substrates into nanocarrier formulations, enabling precise spatiotemporal control over therapeutic interventions and minimizing systemic toxicity [303].
3. **Temperature-Responsive Nanoparticles:** Inspired by thermoresponsive polymers found in nature, researchers have developed temperature-responsive hydrogels for controlled drug delivery applications. These biomimetic hydrogels undergo reversible phase transitions in response to changes in temperature, leading to triggered drug release. By tuning the lower critical solution temperature (LCST) of thermoresponsive polymers, nanocarriers can be designed to release drugs upon exposure to physiological or external heat stimuli, offering on-demand drug delivery with enhanced spatial and temporal control [304]. Heating-induced drug release systems are an innovative and versatile approach in nanomedicine, utilizing various materials that respond to thermal stimuli to achieve controlled and targeted therapeutic delivery. Certain polymers exhibit temperature-sensitive behavior, undergoing phase transitions or changes in solubility at specific temperatures. Polymers like poly(N-isopropylacrylamide) (PNIPAM) transition from a hydrophilic to a hydrophobic state at their lower critical solution temperature (LCST), leading to drug release [305]. These polymeric nanoparticles can be engineered to release drugs when the local temperature is raised. Metal nanoparticles, such as gold nanoparticles and magnetic nanoparticles, can be used to induce localized heating through photothermal or magnetic hyperthermia effects. When these nanoparticles are exposed to laser light (typically near-infrared) or an alternating magnetic field, they generate heat, which can trigger the release of drugs from nanocarriers or directly ablate cancer cells. Mesoporous silica nanoparticles (MSNPs) can be functionalized with thermoresponsive polymers or capped with heat-sensitive molecules. Upon heating, these caps detach or the polymer changes its configuration, allowing the release of the drug molecules stored within the nanopores [306].
4. **Light-Responsive Nanoparticles:** Nature-inspired light-responsive nanocarriers leverage photoreactive molecules or photosensitive materials to achieve spatiotemporal control over drug release using light as an external stimulus. Inspired by photosensitive proteins and pigments in biological systems, such as photoreceptors and chlorophyll, researchers have engineered nanocarriers that undergo photothermal, photochemical, or photophysical transformations upon light exposure, leading to triggered drug release. Light-responsive nanomedicines offer non-invasive, remote-controlled drug delivery modalities with high spatial precision and tunable release kinetics [257].
5. **Magnetic and Ultrasound-Responsive Nanoparticles:** Biomimetic nanocarriers can be engineered to respond to external physical stimuli, such as magnetic fields or ultrasound waves, for triggered drug release. Inspired by the navigational abilities of magnetotactic bacteria and the tissue-penetrating capabilities of ultrasound contrast agents, researchers have developed magnetic and ultrasound-responsive nanomedicines capable of site-specific drug delivery and release under controlled external conditions. Researchers can achieve targeted drug release with spatiotemporal precision and minimal invasiveness by incorporating magnetic nanoparticles or ultrasound-responsive materials into nanocarrier formulations [307].
6. **ROS-responsive nanocarriers**: Oxidative stress (i.e., the imbalance between oxidants and antioxidants in favor of the first ones mentioned) has been found to be elevated in the pathogenesis of many diseases. Thus, another type of stimuli-responsive nanocarriers have been developed—the redox-responsive nanocarriers. The ROS levels are higher in tumor microenvironments than in normal tissues [301].

**Figure 13:**
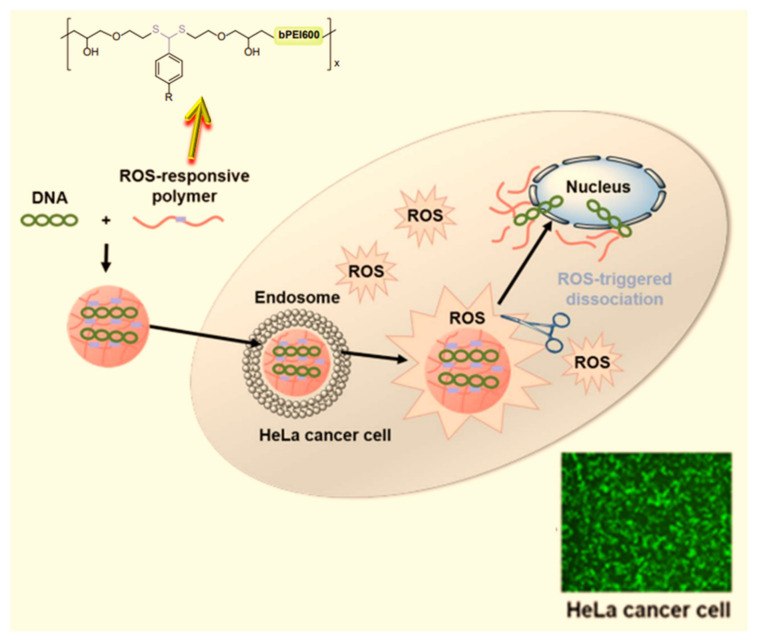
Illustration of intracellular delivery of plasmid DNA to the nucleus of HeLa cancer cell using ROS-responsive polymer-based LWM PEI and thioacetal-linker units (adapted from [308]).

Boronic esters, thioketals, and thioethers are often used to design ROS-sensitive nanocarriers. After internalization by cancer cells, conjugates can disintegrate to release drugs and other components that can subsequently increase ROS and further accelerate the drug release, thus achieving a robust anti-cancer effect [307]. Lin et al. synthesized ROS-responsive materials by ring-opening polymerization between PEI 600 and diepoxide containing stimuli-responsive thioacetal groups [308]. These materials could efficiently condense DNA into stable NPs, which can then be dissociated under ROS-rich conditions for efficient gene delivery (Figure 13).

The three main methods for massively generating ROS are: photodynamic therapy (PDT), sonodynamic therapy (SDT), and enzyme-like catalytic therapy. A recent review highlighted some antibiotic-free antibacterial strategies based on the application of nanomaterials in the above-mentioned three ROS-mediated methods for combating bacteria and biofilms [309].

Bioinspired approaches have paved the way for developing responsive drug release systems in nanomedicines, offering unprecedented control over drug delivery for enhanced therapeutic outcomes. By harnessing the principles of biomimicry, researchers continue to advance the design of nanocarriers with tailored responsiveness to specific biological cues or external stimuli, opening new avenues for precision medicine and personalized therapies [310].

#### 3.4.5. Theranostic Capabilities

Nature-inspired nanomedicines integrate diagnostic and therapeutic functionalities into a single platform, enabling the real-time monitoring of disease progression and on-demand drug delivery [311]. By incorporating imaging agents, biosensors, or contrast agents inspired by biological signaling pathways, these theranostic nanocarriers can provide valuable insights into the disease pathology and treatment response, guiding personalized medicine approaches [312]. Bioinspiration in nanomedicines has led to the development of theranostic platforms with integrated diagnostic and therapeutic capabilities, enabling the simultaneous imaging and treatment of diseases [313]. Drawing inspiration from natural systems, researchers have engineered innovative nanocarriers that combine diagnostic modalities with therapeutic payloads, revolutionizing precision medicine [314]. Here, we explore how bioinspired approaches have been harnessed to design nanomedicines with theranostic capabilities [315].

1. **Multifunctional Nanoparticles:** Inspired by the multifunctionality of biological entities, such as cells and viruses, multifunctional nanoparticles have been developed for theranostic applications. These nanocarriers integrate diagnostic imaging agents, such as contrast agents or fluorescent probes, with therapeutic payloads, allowing for real-time drug delivery and therapeutic response monitoring. By encapsulating imaging agents and drugs within a single nanocarrier platform, researchers can achieve targeted delivery to disease sites while simultaneously visualizing drug distribution and pharmacokinetics *in vivo* [316].
2. **Targeted Imaging and Therapy:** Biomimetic targeting strategies, inspired by the specific interactions observed in biological systems, enable the precise delivery of theranostic agents to diseased tissues or cells. Functionalizing nanocarriers with targeting ligands, such as antibodies or peptides, allows for selective binding to molecular markers overexpressed on diseased cells, enhancing imaging contrast and therapeutic efficacy. By combining targeted imaging with localized drug release, theranostic nanomedicines enable personalized medicine approaches tailored to individual patient profiles, optimizing treatment outcomes while minimizing off-target effects [317].
3. **Responsive Theranostic Platforms:** Nature-inspired stimuli-responsive nanocarriers offer dynamic control over diagnostic imaging and therapeutic release in response to specific physiological cues or external stimuli. Researchers can achieve the triggered release of imaging agents and drugs at disease sites by incorporating stimuli-responsive polymers or nanomaterials, such as pH-sensitive polymers or temperature-sensitive liposomes, enabling spatiotemporal control over theranostic interventions. Responsive theranostic platforms can monitor disease progression in real time and adapt treatment strategies accordingly, enhancing therapeutic efficacy and patient outcomes [318].
4. **Integrated Imaging Modalities:** Inspired by the integrated sensing capabilities of biological organisms, theranostic nanomedicines incorporate a diverse range of imaging modalities for comprehensive disease diagnosis and monitoring. From traditional imaging techniques, such as MRI and PET, to emerging modalities, such as photoacoustic imaging and surface-enhanced Raman scattering (SERS), integrated imaging platforms provide complementary information on disease pathology and treatment response. By combining multiple imaging modalities within a single nanocarrier system, researchers can achieve synergistic diagnostic capabilities and improve the accuracy of disease detection and treatment monitoring [319,320,321,322,323,324].
5. **Personalized Theranostics:** Biomimetic design principles enable the development of personalized theranostic approaches tailored to individual patient characteristics and disease profiles [323]. By leveraging patient-specific imaging data and molecular biomarkers, theranostic nanomedicines can be customized to target specific disease subtypes or molecular signatures, optimizing treatment selection and response prediction [314]. Personalized therapeutics hold promise for guiding precision medicine strategies, enabling the early detection of disease recurrence, facilitating timely therapeutic interventions, and ultimately improving patient outcomes and quality of life [324].

Bioinspired approaches have enabled the development of theranostic nanomedicines with integrated diagnostic and therapeutic capabilities, offering unprecedented opportunities for precision medicine and personalized healthcare. By harnessing the principles of biomimicry, researchers continue to advance the design of multifunctional nanocarriers with tailored theranostic properties, paving the way for transformative innovations in disease diagnosis, treatment, and monitoring. Bioinspiration in nanomedicines offers many opportunities to revolutionize healthcare by leveraging nature’s design principles and evolutionary adaptations. By combining the sophistication of biological systems with the versatility of nanotechnology, bioinspired nanomedicines hold tremendous potential for addressing unmet medical needs, advancing precision medicine, and improving patient outcomes in diverse therapeutic areas.

Biomimetics and bioinspiration are often used in the Green Design of nanopharmaceuticals, an aspect discussed in the following section.

#### 3.4.6. Green Design

Nanopharmaceuticals are essential to human good health and well-being, but they can cause environmental impacts [325]. It is absolutely necessary to take the natural environment into consideration as well as the patient. In this regard, nanopharmaceuticals must be prepared using the **GREENER** concept [**G**: Good Practice for Patients; **R**: Reduced Off-Target Effects and High Specificity; **E**: Exposure Reduction via Fewer Emissions; **E**: Environmental (Bio)degradability; **N**: No PBT (Persistent, Bioaccumulative, and Toxic) Properties; **E**: Effect Reduction (Avoiding Undesirable Moieties); **R**: Risk and Hazard Mitigation], involving the “benign by design” approach [325,326,327]. Thus, the “green” approach by using natural resources/bio-wastes is of real interest today. Special attention is given to plants because they are found in abundance in nature and are an important source of bioactive compounds. These “green biologics”—the plants—are useful for both the production of active principles and the preparation of “green” nanoparticles [328,329]. Kanwar et al. [329] highlighted the importance of using vegetal extracts in the synthesis of NPs, which opened a new era for the development of nontoxic (“green”) methods for the preparation of “green” nanomedicines. Moreover, these authors pointed out the superiority of “green” synthesized NPs from natural and biological sources over chemical synthesized ones. The “green” synthesized NPs are more stable, biocompatible, and eco-friendly than classical synthesized ones. They offer many advantages in drug delivery applications.

## 4. Nanopharmaceuticals in Photo-Based Treatments

Incorporating nanotechnology into biomedicine, namely in the field of oncology, has led to a substantial rise in the number of therapeutic possibilities. Light-mediated therapy, known as phototherapy, including PDT and PTT, is the best option in the treatment of various types of cancer, with a low invasive impact and minimal side effects to normal tissues and organs [330].

PDT employs a photosensitizer (PS) agent to eradicate tumors. This agent is stimulated by light and generates free radicals, ROS, and singlet or triplet oxygen. Consequently, it oxidizes cellular components, causes vascular damage, triggers inflammatory and immune responses, and ultimately results in cell ablation. The primary clinical constraints linked to this therapy include its lack of specificity and targeting, short duration of action, lipophilic nature, and the development of tolerance and resistance in certain malignancies due to the overexpression of glutathione, which counteracts the effects of ROS. To overcome these constraints, one can employ LNP technology to stabilize and solubilize PS, achieve targeted delivery, and co-deliver it with other diagnostic and therapeutic compounds [331].

Attaching pharmaceuticals to a polymeric backbone through covalent bonds is adaptable and allows for precise adjustments. These photosensitive nanomedicines are crafted from a range of polymers featuring diverse architectures, such as linear, branched, and crosslinked structures. The utilization of bioavailability, metabolism, and drug loading allows the inclusion of targeting and triggering components to transport therapeutic molecules to precise sites [332]. The presence of stimulus-responsive monomers within these smart co-polymers provides for the accurate control of drug-release patterns [333]. Light-activated polymers are especially intriguing in this study area because they can combine these advantages with the precise control of medication delivery in space and time. Light may be employed to initiate a multitude of effects. Polymer vehicles undergo several processes, such as morphological changes, cross-linking, bond cleavage, or oxidation through ROS production [334]. In cancer, the intricate characteristics of tumors can impair the effectiveness of therapies that target a single component. However, it is possible to enhance treatment outcomes by simultaneously combining the effects of multiple therapies. Chemo- and photodynamic treatment (PDT) is appealing and has demonstrated encouraging outcomes.

New photosensitizers (PSs) have significantly expanded the scope of phototherapy applications. Current phototherapy techniques primarily utilize exogenous PSs and can be broadly categorized into PDT and PTT [335]. In PDT, PSs generate cytotoxic agents upon light activation, while PTT induces overheating in target tissues. Both modalities lead to apoptotic and necrotic cell death via distinct mechanisms. PDT gained attraction in the 1960s with hematoporphyrin derivatives, culminating in the clinical approval of porfimer sodium (Photofrin) in 1993, primarily for oncological treatments [336]. However, challenges such as poor solubility, aggregation tendencies, and off-target effects have limited the widespread use of PSs. PTT has emerged as a promising cancer treatment due to its short treatment duration and reduced patient discomfort, yet issues remain regarding its targeting efficiency and photothermal conversion efficiency [336,337,338].

Phototherapy, encompassing both PDT and PTT, is under clinical investigation as a treatment avenue for different cancer types. In PDT, photosensitizers trigger the production of singlet oxygen upon light exposure, while PTT utilizes photothermal agents to amplify the laser-induced heating of cells and tissues [338]. Despite PDT’s predominant focus in clinical trials, PTT presents several notable advantages. Unlike PDT, PTT is not reliant on oxygen availability, which is crucial in tumor microenvironments often deprived of oxygen. Additionally, PTT eliminates the need for patients to avoid light exposure to prevent skin toxicity. Various substances, including gold nanoparticles and the small-molecule dye indocyanine green (ICG), have been studied as PTT agents [339]. In contrast to surgical procedures, minimally invasive techniques involve the insertion of small instruments into body cavities, facilitated by flexible optical fiber-bundle devices with diameters typically ranging from 200 to 300 µm. This setup is particularly advantageous for deep-tissue imaging or delivering light for PDT. Furthermore, non-invasive photo-based imaging and therapy within tissues or the body are achievable using near-infrared (NIR) light, specifically in the 650–900 nm range, allowing for deep tissue penetration with minimal attenuation. The adaptability of photo-based imaging and therapy procedures enables easy adjustments according to clinical requirements. Upon intravenous administration, PS-incorporated NPs can be activated by light to induce fluorescence for imaging, generate radical molecules for PDT, or elevate the temperature for PTT [340].

Initially, certain nanoparticles like TiO_2_, ZnO, and fullerenes are employed in PDT as photosensitizing agents by generating singlet oxygen. In contrast, metallic NPs such as AuNPs serve as PSs for PTT by inducing photothermal effects, known as plasmonic PTT. AuNPs efficiently absorb light (photonic energy) and convert it into heat energy. The amount of thermal energy produced by AuNPs depends on the interaction between light and the NPs, facilitated by the surface plasmon resonance effect [341]. Gold nanostructures demonstrate exceptional efficiency in converting light into heat, making them valuable for antibacterial applications. Meeker and colleagues pioneered the creation of polydopamine-coated gold nanocages (AuNCs) specifically tailored for the combined chemo and photothermal treatment of *Staphylococcus aureus* infections [342].

**Figure 14:**
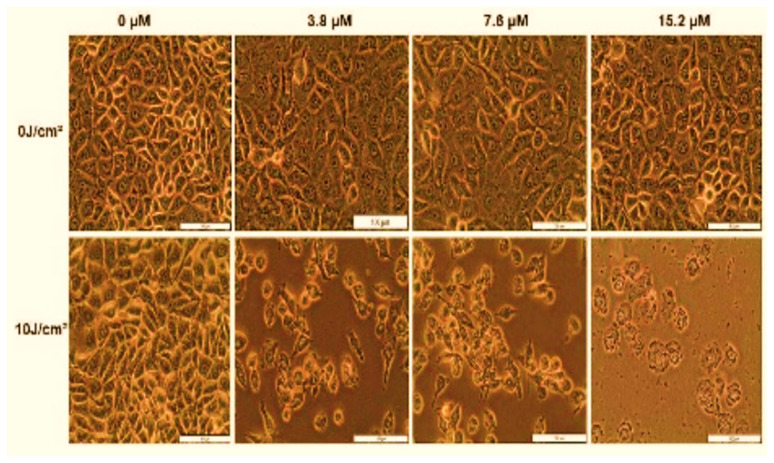
Morphology of MCF-7 breast cancer cells at 12 h post-Hyp-AuNP compound PDT treatment [343].

Mokoena et al. [343] developed a hydrophobic photosensitizer based on Hypericin (a natural bioactive found in *Hypericum species*) physically adsorbed onto AuNPs by sonication. The resulting biohybrid Hyp-AuNPs acted as the PS which improved the effectiveness of PDT and caused MCF-7 breast cancer cell death, mainly by apoptosis. Figure 14 shows the morphological changes of the MCF-7 breast cancer cells treated with the Hyp-AuNP compound and irradiated with a PDT fluence of 10 J/cm^2^, indicating cell death due to PDT treatment.

**Figure 15:**
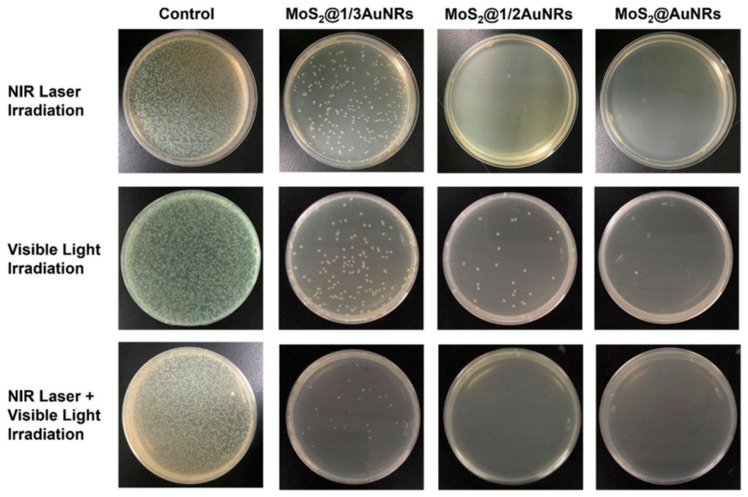
Photographs of the growth of *Escherichia coli* incubated with sterilized water (control) and with three types of composites molybdenum disulfide (MoS_2_)@gold nanorods (AuNRs) on LB agar plates after NIR laser, visible light, or both NIR laser and visible light irradiation [344].

The research group of Yougbaré developed light-responsive nanocomposites based on molybdenum disulfide (MoS_2_) and gold nanorods (AuNRs) which exhibited a remarkable synergistic effect of photothermal therapy and photodynamic therapy, which provides high antibacterial activity against *Escherichia coli* (Figure 15) [344].

The photo-triggered systems encompass photosensitive polymers and anticancer drugs. Typically, light irradiation remotely influences photo-responsive carriers within cancer cells. Initially, optical signals are captured by photochromic molecules (chromophores), which convert photo-irradiation into a chemical signal via a photoreaction, ultimately triggering drug release through changes in carrier structures [345]. This photo-triggered system exploits the photo-responsive chemistry, including photoisomerization utilizing azobenzene (AZO), spiropyran (SP), and dithienylethene (DTE), photo-induced rearrangement employing 2-diazo-1,2-naphthoquinone (DNQ), photo-based cleavage utilizing o-nitrobenzyl ester, coumarinyl ester, and pyrenylmethyl ester, and photo-induced energy conversion utilizing AZO derivatives [346].

In recent times, quantum dots (QDs-Ge-QDs, Ag2S QDs, CdS, CdSe, PbSe, InP, CdTe, and tungsten sulfide (WS2) QDs) have surged in popularity due to their enhanced fluorescence properties compared to conventional dyes [346]. These properties include size-dependent fluorescence, narrow emission spectra, and resistance to photo-bleaching [347].

Single-walled carbon nanohorns (SWCNHs) represent horn-shaped nanostructure aggregates derived from graphene, boasting a range of versatile qualities that make them ideal candidates for targeted drug delivery systems. They exhibit ease of synthesis and functionalization, allowing for the acquisition of desired physicochemical properties. Moreover, no adverse toxicological effects have been reported following *in vivo* administration, further enhancing their appeal for medical applications [348].

Numerous semiconducting materials (such as copper-, bismuth-, and tungsten-chalcogenides; MoS2; black P; Ag2S; WO3, etc.) have been proposed as PTT agents. This group typically exhibits exceptional near-infrared absorption properties and photothermal conversion efficiency (PCE), although their long-term biocompatibility remains largely untested. The second group comprises oxide materials, including both iron- and manganese-based variants. The PTT performance of these materials often lags behind that of the former group due to their inferior intrinsic PCE. However, their comparative disadvantage is offset by the extensive research conducted on them for biomedical applications. Some oxide materials, such as iron oxides, have already received approval for human use, underscoring their presumed high safety and biocompatibility [349].

Additionally, membrane-camouflaged nanoparticles may possess inherent biological functionalities derived from the membrane source, such as targeting ligands, cell adhesion molecules, or immunomodulatory proteins, which can further enhance their therapeutic efficacy or diagnostic capabilities [349,350]. Spherical vesicles with unique capabilities, with curcumin (CUR) that was encapsulated within stealth liposomes made of 1,2-distearol-sn-glycero-phosphoethanolamin-N-(poly[ethylene glycol], upon exposure to LED light (2.5 J/cm²), demonstrated significant photocytotoxicity against squamous cell carcinoma (SCC-25) and melanoma skin cancer cells (MUG-Mel2), resulting in apoptosis rates of 40% and 30%, respectively [351,352].

Zein, known for its excellent self-assembly properties, can effectively host photosensitizers as well. Targeted multifunctional nanoparticles comprising zein/hyaluronic acid (HA)/tannin (TA)/Cu^2+^ loaded with IR780 (ZHTC@IR780) were developed for synergistic cancer therapy via chemo-dynamic therapy (CDT) and PDT. Experimental evidence suggests that ZHTC@IR780 nanoparticles can alleviate the tumor hypoxic microenvironment by catalyzing the decomposition of endogenous H_2_O_2_ into O_2_, which further reacts with O_2_ to generate toxic ^1^O_2_ upon 808 nm laser irradiation. The glutathione oxidase-like effects of ZHTC@IR780 NPs lead to the generation of Fenton-like Cu^+^ ions and the depletion of GSH, resulting in efficient hydroxyl radical (•OH) production. Moreover, the combination of CDT and PDT enhances the antitumor effect, with PDT inducing immunogenic cell death, increasing calreticulin eversion, releasing histones with high mobility, and promoting the apoptosis of tumor cells [352].

## 5. NanoTheranostic Agents

Theranostic agents refer to a class of compounds or substances that have both therapeutic and diagnostic capabilities. The term “theranostics” is a combination of “therapy” and “diagnostics”. These agents play a crucial role in personalized medicine, as they allow for the simultaneous monitoring of a medical treatment’s effectiveness and the delivery of targeted therapy. The integration of diagnosis and therapy into a single agent can improve treatment outcomes, reduce side effects, and optimize the overall therapeutic process [353,354]. The properties of theranostic agents include: diagnostic capabilities, therapeutic capabilities, personalized medicine, real-time monitoring, multimodal imaging, etc.

*Diagnostic Capabilities*: Theranostic agents often contain components that enable the imaging or detection of specific biomarkers associated with a particular disease. This diagnostic aspect helps in identifying the location, extent, and characteristics of the disease [354,355].

*Therapeutic Capabilities*: In addition to their diagnostic functions, theranostic agents have therapeutic components designed to deliver targeted treatment. This could involve drugs, nanoparticles, or other therapeutic agents that are tailored to act on specific cells or tissues [356,357].

*Personalized Medicine*: The use of theranostics is aligned with the principles of personalized medicine, where treatments are tailored to individual patients based on their specific molecular or genetic characteristics. This approach aims to maximize treatment efficacy while minimizing adverse effects [358,359].

*Real-time Monitoring*: Theranostic agents enable the real-time monitoring of the treatment response. This is valuable for adjusting treatment plans as needed, ensuring that the therapy remains effective over time [360,361].

*Multimodal Imaging*: Many theranostic agents incorporate multiple imaging modalities, such as positron emission tomography (PET), single-photon emission computed tomography (SPECT), magnetic resonance imaging (MRI), or optical imaging. This allows for more comprehensive and accurate diagnostic information [362].

Examples of theranostic agents include nanoparticles loaded with both therapeutic drugs and imaging agents, radioactive tracers conjugated to therapeutic molecules, and molecular probes that can identify specific biomarkers and deliver therapeutic payloads [363]. The development of theranostic agents is a rapidly evolving field, and ongoing research continues to explore new ways to integrate diagnostic and therapeutic functions for various medical conditions, including cancer, cardiovascular diseases, and neurological disorders.

The nanoparticles (NPs) are most preferred as theranostic agents as they offer many advantages compared to larger particles, such as an increased surface-to-volume ratio, which provides numerous active sites for bio-reactions. The most commonly used nano-theranostics materials are dyed nanoparticles (nano-pigments), metal nanoparticles, liposomes, and carbon nanomaterials [364,365]. Mirahadi et al. described how lipid-based nanoparticles enhance the efficiency and precision of diagnostic methods, allowing for the improved imaging and targeted delivery of diagnostic agents in medical applications [365].

**Figure 16:**
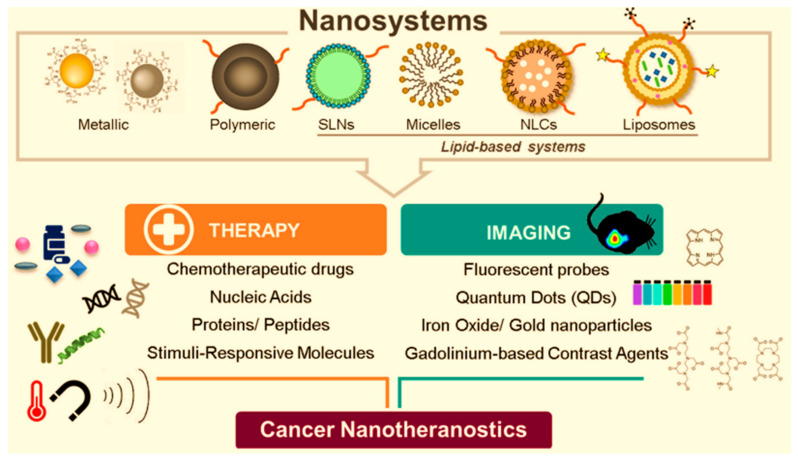
Nanotheranostics: metallic, polymeric, and lipid-based nanosystems for cancer management [366].

Figure 16 illustrates the main nanosystems (metallic, polymeric, and lipid-based) used as nanotheranostics for cancer management [366].

**Figure 17:**
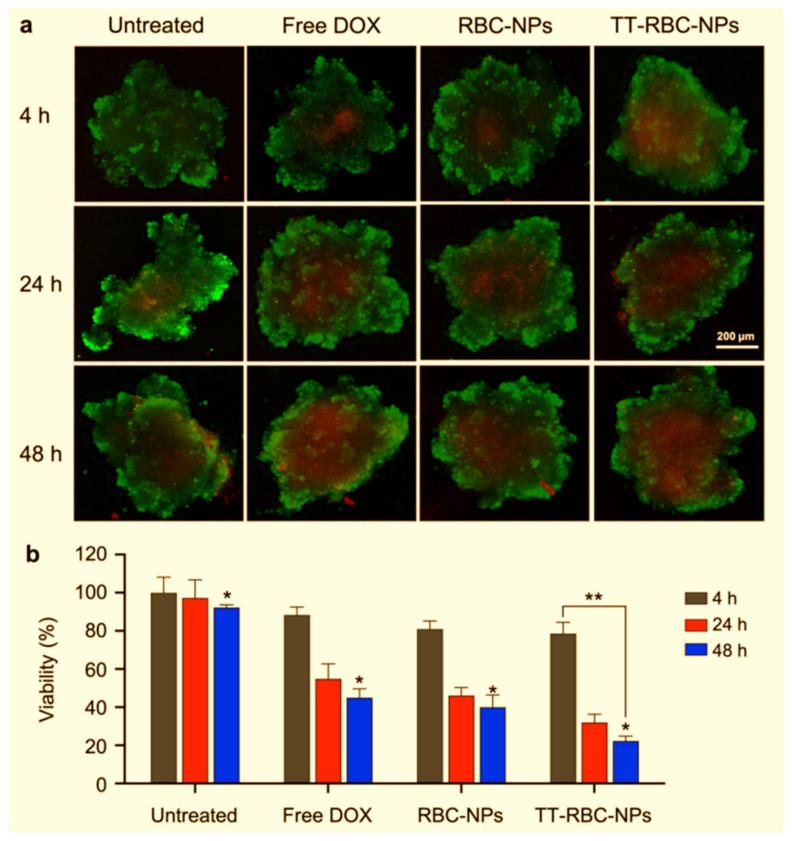
*In vitro* cellular toxicity of TT-RBC-NPs in three-dimensional (3D) spheroids. (**a**) *In vitro* live/dead cell imaging of tumor spheroid, untreated and treated with free DOX, RBC-NPs, and TTRBC-NPs (equivalent of 1 µg/mL DOX) for a period of 4, 24, and 48 h. Two-color fluorescence, live (green channel) and dead (red channel), enables evaluation of live and dead cells to determine cell viability. Scale bar = 200 µm. (**b**) *In vitro* cytotoxicity study using CellTiter-Glo^®^ 3D cell assay of untreated, free DOX, RBC-NPs, and TT-RBC-NPs against 3D MCF-7 spheroids. Results represent mean ± SD (*n* = 3). * the significance between Untreated, Free DOX, RBC-NPs, and TT-RBC-NPs at 48 h incubation (*p* < 0.05). ** the significance between 4 h and 48 h incubation of TT-RBC-N [367].

Human red blood cell (RBC) membrane-cloaked PLGA nanoparticles (TT-RBC-NPs) loaded with doxorubicin (DOX) and that have enhanced targeting functionality were designed as a targeted nanotheranostic against MCF-7 breast cancer cells [367]. This biomimetic drug delivery system was capable of not only specifically binding to targeted MCF-7 cancer cells, effectively delivering DOX, but also visualizing the targeted cancer cells. Figure 17 displays the *in vitro* cellular toxicity of TT-RBC-NPs in 3D MCF-7 spheroids.

## 6. Challenges and Limitations of Nanopharmaceuticals

Nanopharmaceuticals, which involve the use of nanomaterials for medical purposes, have shown great promise in various medical applications, including drug delivery, imaging, and diagnostics. However, like any emerging technology, nanopharmaceuticals come with their own set of limitations, challenges, and potential toxicity concerns. Some of the key issues associated with nanopharmaceuticals are biocompatibility and toxicity, immune system interactions, biodistribution and accumulation, regulatory challenges, environmental impacts, ethical and societal concerns, long-term effects and chronic toxicity, etc.

Nanopharmaceuticals may exhibit different biological behaviors compared to larger counterparts. Their small size can lead to unique interactions with biological systems that may result in unexpected toxic effects [368]. Their blood compatibility is significantly influenced by diverse properties of the material surface. The interaction between blood and the material is contingent upon physicochemical characteristics, including the surface area, surface charge, hydrophobicity/hydrophilicity, and other relevant factors. In the case of nanoparticles, the critical determinants in these interactions are the size effect, structure, and surface properties [369].

Some nanomaterials may cause toxicity due to factors such as their composition, surface charge, and ability to generate ROS [370,371]. Long-term exposure studies are often lacking, making it challenging to assess the chronic toxicity of nanopharmaceuticals [372]. Nanoparticles may interact with the immune system in ways that are not fully understood [373]. This interaction could lead to immunotoxicity, inflammation, or other adverse effects. The immune system may recognize nanomaterials as foreign entities, potentially leading to immune responses that could compromise the intended therapeutic effects [374].

Nanopharmaceuticals may have different biodistribution patterns compared to conventional drugs [375]. Their accumulation in certain organs or tissues could raise concerns about long-term effects on those specific areas. Clearance mechanisms for nanomaterials are not always well-defined, and the persistence of nanoparticles in the body could lead to unintended effects. The regulatory framework for nanopharmaceuticals is still evolving, and there may be gaps in assessing their safety and efficacy [376,377]. Standardized testing methods and criteria for evaluating nanomaterials used for nanopharmaceutics are needed to ensure consistent regulatory oversight. The lack of standardized characterization techniques makes it challenging to compare results across different studies and assess the safety of various nanomaterials [378].

The disposal of nanopharmaceuticals and their potential release into the environment can raise environmental concerns. The impact of nanomaterials on ecosystems and their potential to accumulate in the environment are areas of ongoing research [379,380]. There are ethical considerations regarding the use of nanopharmaceuticals, especially with regard to potential unknown risks and long-term effects [381,382]. Public perception and acceptance of nanotechnology in medicine may influence the adoption of nanopharmaceuticals. Long-term studies assessing the chronic toxicity of nanomedicines are often limited, making it challenging to predict the effects of prolonged exposure [383]. The persistence of nanoparticles in the body raises concerns about their potential to cause cumulative toxicity over time.

Researchers and regulatory agencies continue to address these challenges through ongoing studies, improved testing methodologies, and the development of guidelines for the safe use of nanopharmaceuticals. It is crucial to balance the potential benefits of nanopharmaceuticals with a thorough understanding of their risks to ensure their safe and effective application in medical practices. The goal is to harness the potential benefits of nanotechnology in pharmacy as well as medicine, while minimizing the risk of adverse effects.

**Figure 18:**
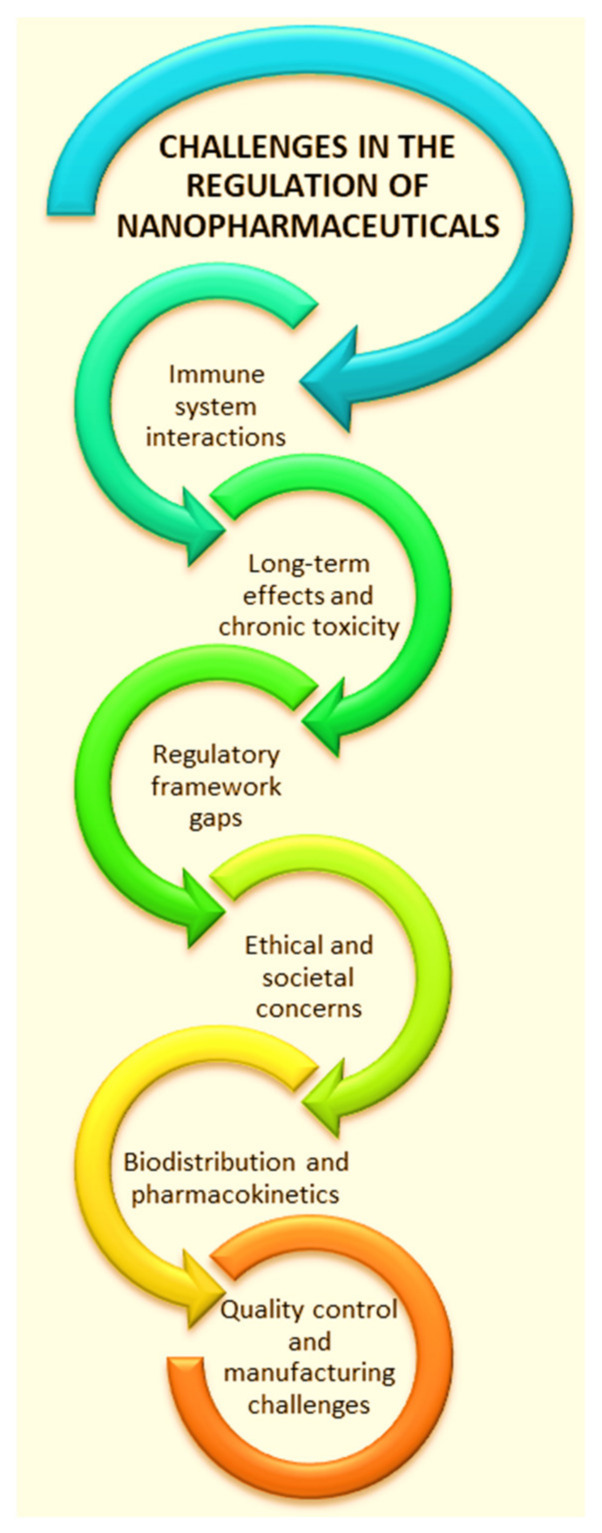
Schematic representation of the main challenges regarding nanopharmaceuticals. Figure was created Microsoft PowerPoint.

Figure 18 displays the main challenges regarding nanopharmaceuticals.

Medicines represent an advantage for humanity in combating diseases, although not fully, but to a great extent. Along with the beneficial effects, they can also present some side effects, sooner or later [384]. Moreover, randomly throwing away medicines leads to environmental pollution. Thus, a new science was born—**ecopharmacovigilance**—which is dealing with the detection, assessment, understanding, and prevention of adverse effects or other problems concerning the presence of pharmaceuticals in the environment, which affect both humans and animals [385,386,387]. The term “ecopharmacovigilance” was used for the first time by Velo in 2007 [388].

Therefore, it is our responsibility to keep the environment clean for the safety of the next generations of people. The notions of sustainability and *Green Chemistry*/*Green Nanotechnology* must be introduced into education, in the *curriculum* of higher schools and faculties. Other solutions to reduce the environmental impact of nanopharmaceuticals are: (i) the use of green strategies by adopting the Green Chemistry principles for the development of “green” nanopharmaceuticals; (ii) the preparation of biodegradable NPs; (iii) the use of living mater and the valorization of bio-wastes for the development of nano-biopharmaceuticals; and (iv) improving regulations and guidelines for pharmaceutical rational pharmaco-derived waste management.

## 7. Nanopharmaceuticals’ Testing

Before being used, medicines must be tested to have the safety guarantee. Animals are commonly used for this purpose. But some questions are asked: *Are animal tests quite conclusive, considering the physiological differences between humans and animals*? *Is it ethical to perform tests on animals*? There are many problems regarding translation from animal experimentation results to humans including (i) genetic and physiological differences between species, and (ii) differences between human diseases and animal models of diseases [389].

Moreover, the tests on animals are expensive and time-consuming, and involve animal suffering. Therefore, alternative strategies have been developed to avoid animal use. In 1992, Russell and Burch defined these alternatives by **three R’s**: *Reduction* (the use of the minimum number of animals), *Refinement* (related to reducing the pain, discomfort, and distress of animals during procedures), and *Replacement* (the use of non-animal living systems, non-living systems, and computer simulations) [390,391]. Later, in 1995, Banks introduced **the 4th R**—*Responsibility* (dealing with the integrity, honesty, and scientific correctness in the animals´ use in research tests) [392].

As alternative strategies avoiding animals, we mention the usage of:

(i) *In vitro* cell and tissue cultures—routinely used for the preliminary screening of potential drug toxicity and involves the *in vitro* growth of animal/human cells outside the body in the laboratory environment;
(ii) Microbiological systems such as prokaryotes (e.g., *Escherichia coli*) and fungi (e.g., *Saccharomyces cerevisiae*, *Aspergillus nidulans*);
(iii) Stem cells—toxicological tests involving the insertion of disease genes into embryonic stem cells, which then differentiate into human disease tissues that can be further used for drug screening;
(iv) Invertebrate organisms—such as *Drosophila melanogaster*, Hydra, and others;
(v) Lower vertebrates such as zebrafish (*Danio rerio*)—an attractive option due to the genetic relatedness to higher vertebrates including mammals;
(vi) Plant-tissue based material—studies on the effect of nanopharmaceuticals on the environment performed on *Brassica juncea*, resulting in drug-induced defense responses and the activation of the detoxification mechanism;
(vii) DNA chips—glass slides with an array of genes or DNA fragments that enable the pharmacogenetics studies;
(viii) Microfluidics—a series of tiny compartments each containing tissue samples (originating from different body parts) and linked by microchannels through which flows a blood substitute in which the test drug it is introduced;
(ix) Computer analysis models—*in silico* experiments on computer-generated simulations by using various softwares such as the Computer-Aided Drug Design (CADD, used to predict the receptor-binding site for a potential drug) and the Quantitative Structure–Activity Relationship (QSAR, used to predict the carcinogenicity);
(x) Chitosan films—used as a substitute for animal and human cadaver epidermal sheets used for testing the *in vitro* permeation of polar and nonpolar drugs [393,394].

The use of plant tissues to test the effect of nanopharmaceuticals on living systems is an attractive “green” approach. Thus, decellularized plant-derived scaffolds are a sustainable alternative to animal-derived sources. They have been used in drug testing due to their natural fluidic vascularized transport system (with micro-vessels and fine capillaries), which resembles that of the branching mammalian vascular network [395]. Predeina et al. developed a simple, bioinspired model for the *in vitro* testing of nanopharmaceutical drugs based on magnetic nanoparticles and used it in thrombosis treatment [396]. Their model was based on a vasculature scaffold of decellularized spinach baby leaves that enables the simple, optical control of the magnetic nanoparticles´ behavior in the spinach branched system, which mimics human vasculature.

## 8. Future Directions and Concluding Remarks

This study presents current issues related to the application of nanotechnology in pharmaceutical and biomedical fields. Properties of nanocarriers such as their size, shape, surface charge, and rigidity of structure need to be studied in detail to develop an efficient nanosystem that can safely deliver therapeutic drug concentrations effectively.

“Green” aspects were also highlighted, such as: biomimetics/bioinspiration/bioderivation, the use of modern and clean methods for NPs’ preparation with a low cost and minimal energy consumption, avoiding the use of toxic substances.

Current trends in the modern design of nanopharmaceuticals were pointed out. Attractive properties can be provided, especially by bionanoformulations. For example, biocompatibility and stealth properties can be assured through biomimetic and bio-inspired strategies like coating NPs with artificial or natural cell membranes—camouflage that makes nanopharmaceuticals more attractive in bioapplications. Moreover, further surface modifications with RGD peptide or with folic acid allow them to target at the tumor site.

One of the most modern trends in NPs manufacturing—3D printing—has been presented. In the near future, 3D printing in nanopharmaceutical development will continue its evolution as a cornerstone in precision medicine and advanced manufacturing. Emerging trends, including integrating artificial intelligence and machine learning, hint at the exciting possibilities. This review concludes with a synthesis of critical insights, emphasizing the imperativeness for continued research and innovation to unlock the full potential of 3D printing in shaping the future of nanopharmaceuticals. As personalized medicine and patient-specific formulations continue to revolutionize healthcare, exploring future directions and summarizing key insights are essential for advancing the field and realizing its full potential. This section delves into the future directions of personalized medicine and patient-specific formulations, highlighting emerging trends, challenges, and opportunities for innovation. One of the most promising avenues for advancing personalized medicine is integrating multi-omics data, including genomics, transcriptomics, proteomics, metabolomics, and microbiomics. By combining insights from multiple omics domains, researchers can comprehensively understand disease mechanisms, identify novel biomarkers, and develop targeted therapies tailored to individual patient profiles. Future research efforts should leverage advanced bioinformatics tools and machine learning algorithms to analyze multi-omics data and translate findings into clinically actionable insights. As a disruptive force in drug development, 3D printing technology promises to redefine the boundaries of precision medicine, offering tailored treatment regimens and patient-specific formulations that herald a new era in healthcare.

Nanomedicine holds immense promise for revolutionizing drug delivery and therapy in personalized medicine. Future directions in nanomedicine include the development of multifunctional nanoparticles, targeted drug carriers, and theranostic agents capable of simultaneous drug delivery and diagnostic imaging. By harnessing the unique properties of nanoparticles, such as size-dependent pharmacokinetics, surface functionalization, and controlled drug release, researchers can design patient-specific formulations with enhanced therapeutic efficacy and reduced systemic toxicity. Digital health technologies, including wearable devices, mobile health apps, and remote monitoring systems, are poised to transform personalized medicine. These technologies enable the continuous monitoring of patient health parameters, real-time data collection, and personalized feedback, facilitating early disease detection, treatment optimization, and patient engagement. Future directions in digital health include the integration of artificial intelligence algorithms, predictive analytics, and personalized decision support tools to empower patients and healthcare providers in making informed treatment decisions. As personalized medicine evolves, regulatory considerations and policy frameworks will be critical in ensuring patient safety, efficacy, and access to innovative therapies. Future directions in regulation include the development of adaptive pathways, expedited review processes, and value-based reimbursement models tailored to the unique characteristics of personalized therapies. Regulatory agencies must collaborate with healthcare stakeholders, industry partners, and patient advocacy groups to establish clear guidelines, streamline regulatory pathways, and foster innovation while safeguarding public health.

Addressing personalized medicine’s ethical, legal, and social implications is essential for fostering public trust, equity, and access to healthcare. Future directions in ethics and governance include the development of guidelines for informed consent, data privacy, and equitable access to personalized therapies. Stakeholders must engage in proactive dialogue, community outreach, and education initiatives to raise awareness of personalized medicine’s benefits and challenges and ensure its benefits are equitably distributed across diverse populations.

Personalized medicine and patient-specific formulations represent a paradigm shift in healthcare, offering the promise of tailored treatments, improved patient outcomes, and enhanced quality of life. By embracing emerging technologies, advancing regulatory frameworks, and addressing ethical considerations, stakeholders can accelerate the adoption of personalized medicine and realize its transformative potential in revolutionizing healthcare delivery. As we navigate the complex landscape of personalized medicine, collaboration, innovation, and a commitment to patient-centered care will be paramount in shaping the future of healthcare and ushering in a new era of precision medicine.

Photo-based nanomedicines have proven efficacy in both imaging and therapeutic applications. However, when selecting light for use in diagnostics and therapies, it is crucial to weigh the associated risks and benefits carefully. Thanks to significant advancements in nanotechnology, photosensitive nanomedicines can now be tailored for various photo-based diagnostics and therapies, including photo-triggered systems, nanoparticles containing photosensitizing agents, and NPs acting as PSAs themselves.

It is imperative that the efforts of scientists from various fields unite in order to find new green, economic solutions based on clean nanotechnologies for the ecological production of new pharmaceutical nanoformulations with a high bioperformance, thus ensuring the improvement of the quality of people’s lives and those of other living things, and also protecting the environment. Last but not least, it must be stated that educating the population from a young age, regarding the adoption of green solutions in all areas, is of overwhelming importance in this regard. When choosing pre-cursor materials for the green development of nanopharmaceuticals, the use of natural raw materials and the valorization of bio-wastes must be in the spotlight of the scientific research because they are low-cost, highly abundant/available, and provide interesting features such as biodegradability, bio-activities, and eco-friendliness. Plants are a valuable raw material as they are found in abundance in nature and they must be exploited in the pharmaceutical field, being a rich source of bioactive compounds that can be used in the development of nano-biopharmaceuticals. Also, plants are bionanofactories for the green synthesis of nanoparticles that can be successfully applied in the biomedical field.

## Figures and Tables

**Table 1:** Nanomicelles’ applications for supporting human health.

Active Principle Loaded in Nanomicelles	Applications	References
Casein nanomicelles	Nutraceutics, pharmaceutics, and cosmetics	[23,24,25]
Cholesteryl succinyl silane nanomicelles with doxorubicin and gold nanoshells	Cancer therapy	[26,27]
Polyplex nanomicelles	CNS therapeutics, Muscle-Targeted mRNA Delivery	[28,29,30]
Indisulam stabilized in DSPE-PEG_2000_ micelles + PC	Cancer therapy	[31,32,33]
Cyclosporine A containing nanomicelles using a polyvinyl caprolactam-polyvinyl acetate-polyethylene glycol (PVCL-PVA-PEG) graft copolymer	Topical delivery system for ocular administration	[34,35,36,37]
Curcumin nanomicelles	Asthenoteratozoospermia therapy, recurrent aphthous stomatitis treatment	[38,39,40]
Silymarin, soluplus nanomicelles with d-α-tocopherol polyethylene glycol 1000 succinate	Antioxidant therapeutics	[41,42]
β-carotene-loaded chitosan-graft-poly(lactide) nanomicelles	Functional food, cosmetics, and antioxidant therapeutics	[43,44]
Resveratrol-loaded amphiphilic bioconjugate nanomicelles	Colorectal cancer therapeutics	[45,46]
Celastrol-loaded nanomicelles	Corneal neovascularization therapeutics	[47,48]

**Table 2:** Metallic nanoparticles (MNPs) used in different biomedical applications.

MNPs/MO NPs	Bioactivity/Bio-Applications	References
**Fe_2_O_3_NPs**	Cancer imaging and treatment, antimicrobial and antioxidant activities	[108,109,110]
**CuO NPs**	Antimicrobial, anticancer, antioxidant, anti-inflammatory, and antidiabetic activities	[111,112,113]
**AuNPs**	Cancer diagnosis, PDT, PTT, gene/drug delivery systems, and antiviral and antimicrobial agents	[113,114,115,116,117]
**ZnO NPs**	Antimicrobial and anticancer agents	[77,118]
**PdNPs**	Photothermal therapy, antimicrobial, antioxidant, antidiabetic, and anticancer therapy	[119,120,121]
**SeNPs**	Supplementation, immunostimulatory effect in cancer therapies; anticancer and antioxidant activities	[122,123,124]
**PtNPs**	ROS scavenger, neuroprotective effects, anti-colitis agent, powerful antioxidant, drug/gene delivery, and cancer therapy	[85,86,125,126,127,128,129]
**CeO_2_ NPs**	Antioxidant activity, multiple sclerosis therapeutic, and wound healing agent	[130,131,132,133,134]
**TiO_2_ NPs**	Antibacterial effect, PDT, and drug delivery	[135,136,137]
**AgNPs**	Antimicrobial, anticancer and anti-inflammatory activity, drug delivery, tissue regeneration, and healthcare products	[138,139,140,141]

## Abbreviations and Symbols

AgNPs	Silver nanoparticles
AuNCs	Gold nanocubes
AuNPs	Gold nanoparticles
AuNRs	Gold nanorods
AuS	Gold shell
AZO	Azobenzene
Bac	*Bifidobacterium*
BBB	Blood–Brain Barrier
BSA	Bovine serum albumin
CAD	Computer-aided design
CBNs	Carbon-based nanomaterials
CDT	Chemo-dynamic therapy
CeO_2_ NPs	Cerium oxide nanoparticles
Chl	Chlorophyll
Ce6	Chlorin e6
CMC	Critical micellar concentration
CNMs	Carbon nanomaterials
CNHs	Carbon nanohorns or carbon nanocons
CNS	Central nervous system
CNTs	Carbon nanotubes
CS	Chitosan
CSNCs	Chitosan nanocapsules
CT	Computed tomography
CuO NPs	Copper-oxide nanoparticles
CUR	Curcumin
DDS	Drug Delivery System
DLS	Dynamic Light Scattering
DNA	Deoxyribonucleic acid
DNPs	Diatom nanoparticles
DoE	Design of experiments
DOX	Doxorubicin
DPA-QDs	D-penicillamine-coated quantum dots
DWCNTs	Double-walled carbon nanotubes
EM	*E. coli*-derived membrane
EMA	European Medicines Agency
EO	Essential oils
EPR	Enhanced permeability and retention effect
EVs	Extracellular vesicles
FDA	United States Food and Drug Administration
Fe_2_O_3_NPs	Iron-oxide nanoparticles
FTIR	Fourier-transform infrared spectroscopy
5-FU	5-Fluorouracil
GI	Gastrointestinal
GLP-1	Glucagon-like peptide 1
GSH	Glutathione
HspG41C	Mutant heat-shock protein (Hsp) cage, in which the Glycine from position 41 was replaced by a Cysteine residue
Hyp-AuNP	Gold Nanoparticles conjugated with Hypericin
ICG	Indocyanine green dye
ICH	International Council for Harmonization of Technical Requirements for Pharmaceuticals for Human Use
ICG	Indocyanine green dye
JNK	c-Jun N-terminal kinase
LB	Luria Bretani agar
LCP	*L. casei* postbiotics
LCST	Lower critical solution temperature
LNPs	Lipid nanoparticles
MHAp	Magnetic hydroxyapatite
MHNRs	Magnetic hydroxyapatite nanorods
MHT	Magnetic hyperthermia
MNPs	Metallic nanoparticles
MOFs	Metal–organic frameworks
MONPs	Metal-oxide nanoparticles
MoS_2_@AuNR	Gold Nanorod-Decorated Metallic MoS_2_ Nanosheets
MPFVs	Minimally processed fruits and vegetables
MRI	Magnetic resonance imaging
MSNPs	Mesoporous silica nanoparticles
MUG-Mel2	Melanoma skin cancer cells
MWCNTs	Multi-walled carbon nanotubes
NDFs	Nanoscale dietary fibers
NGPs	Next-generation probiotics
NIR	Near-infrared
NLCs	Nanostructured lipid carriers
NPs	Nanoparticles
NRF2	Nuclear factor erythroid 2–related factor 2
NTs	Nanotubes
OMV	Outer membrane vesicles
OVA	Ovalbumin
P38 MAPK	p38 mitogen-activated protein kinases
PAA	Poly(acrylic acid)
PAA-Pt	Poly(acrylic acid) (PAA)-protected platinum nanoparticle species
PBT	Persistent, Bioaccumulative, and Toxic
PCE	Photothermal conversion efficiency
PCL	Poly(ε-caprolactone)
PDT	Photodynamic therapy
PdNPs	Palladium Nanoparticles
PEG	Poly(ethylene glycol)
PET	Positron Emission Tomography
PLA	Poly(lactic acid)
PLGA	Poly(lactic-co-glycolic acid)
P127	Pluronic F127
PNIPAM	Poly(N-isopropylacrylamide)
PS	Photosensitizer
PtNPs	Platinum nanoparticles
PTT	Photothermal therapy
PVA	Poly(vinyl alcohol)
QDs	Quantum dots
RBCs	Red Blood Cells
RGD	Arginyl-glycyl-aspartic acid (Arg-Gly-Asp, R: arginine; G: glycine; D: aspartic acid)
RNA	Ribonucleic acid
ROS	Reactive Oxygen Species
RWE	Real-world evidence
SCC-25	Squamous cell carcinoma
SCFA	Short-chain fatty acids
SEM	Scanning Electron Microscopy
SeM	Diselenide-bridged mesoporous silica nanoparticles
SeNPs	Selenium nanoparticles
SERS	Surface-enhanced Raman scattering
SLA	Stereolithography
SLNs	Solid lipid nanoparticles
SLS	Selective laser sintering
SNEDDS	Self-nanoemulsifying drug delivery systems
SOD	Superoxide dismutase
SPECT	Single-photon emission computed tomography
srNPs	Stimuli responsive nanoparticles
SUVs	Small unilamellar lipid vesicles
SWCNTs	Single-walled carbon nanotubes
SWCNHs	Single-walled carbon nanohorns
TiO_2_ NPs	Titanium oxide nanoparticles
UC	Ulcerative colitis
ZHTC@IR780	Zein/hyaluronic acid (HA)/tannin (TA)/Cu^2+^ loaded with IR780
ZnO NPs	Zinc-oxide nanoparticles
